# The Role of Serotype Interactions and Seasonality in Dengue Model Selection and Control: Insights from a Pattern Matching Approach

**DOI:** 10.1371/journal.pntd.0004680

**Published:** 2016-05-09

**Authors:** Quirine A. ten Bosch, Brajendra K. Singh, Muhammad R. A. Hassan, Dave D. Chadee, Edwin Michael

**Affiliations:** 1 Department of Biological Sciences, University of Notre Dame, Notre Dame, Indiana, United States of America; 2 Hospital Sultanah Bahiyah, Alor Setar, Kedah, Malaysia; 3 Department of Life Sciences, University of the West Indies, Saint Augustine, Trinidad and Tobago; Santa Fe Institute, UNITED STATES

## Abstract

The epidemiology of dengue fever is characterized by highly seasonal, multi-annual fluctuations, and the irregular circulation of its four serotypes. It is believed that this behaviour arises from the interplay between environmental drivers and serotype interactions. The exact mechanism, however, is uncertain. Constraining mathematical models to patterns characteristic to dengue epidemiology offers a means for detecting such mechanisms. Here, we used a pattern-oriented modelling (POM) strategy to fit and assess a range of dengue models, driven by combinations of temporary cross protective-immunity, cross-enhancement, and seasonal forcing, on their ability to capture the main characteristics of dengue dynamics. We show that all proposed models reproduce the observed dengue patterns across some part of the parameter space. Which model best supports the dengue dynamics is determined by the level of seasonal forcing. Further, when tertiary and quaternary infections are allowed, the inclusion of temporary cross-immunity alone is strongly supported, but the addition of cross-enhancement markedly reduces the parameter range at which dengue dynamics are produced, irrespective of the strength of seasonal forcing. The implication of these structural uncertainties on predicted vulnerability to control is also discussed. With ever expanding spread of dengue, greater understanding of dengue dynamics and control efforts (*e*.*g*. a near-future vaccine introduction) has become critically important. This study highlights the capacity of multi-level pattern-matching modelling approaches to offer an analytic tool for deeper insights into dengue epidemiology and control.

## Author Summary

The fluctuations of multi-serotype infectious diseases are often highly irregular and hard to predict. Previous theoretical approaches have attempted to disentangle the drivers that may underlie this behaviour in dengue dynamics with variable success. Here, we examine the role of such drivers using a pattern-oriented modelling (POM) approach. In POM, multiple patterns observed at different scales are used to test a model’s proficiency in capturing real-world dynamics. We examined dengue models with combinations of cross-immunity, cross-enhancement, seasonal fluctuations in the transmission rate, and with sensitivity analyses of asymmetric transmission rates between serotypes as well as the possibility for four subsequent heterologous infections. We demonstrate the ability of POM to model dynamical drivers that have gone unnoticed in single pattern or synthetic likelihood approaches. Further, our results present a determining role of seasonality in the selection and operation of these processes in governing dengue dynamics, in particular when full, heterologous immunity is assumed to occur after a secondary infection. We show that this structural model uncertainty can have important practical significance, as demonstrated by the differences in control efforts required to disrupt transmission. These results highlight the importance of localised model selection and calibration using multiple data-matching, as well as taking explicit account of model uncertainty in predicting and planning control efforts for multi-serotype diseases.

## Introduction

With a 30-fold increase in incidence over the last five decades, dengue poses an increasing threat to about two thirds of the world population [1]. Dengue, caused by a group of viruses belonging to the *Flavivirus* genera, circulates in four major serotypes (DENV 1–4) [2], and manifests in a wide spectrum of clinical forms, from subclinical to classic dengue fever to the more serious forms of the disease, namely, dengue haemorrhagic fever (DHF) and dengue shock syndrome (DSS). In the absence of treatment, dengue can be highly fatal in subjects with DHF or DSS, with a case-fatality rate of 15%, which may be reduced to 1% with adequate medical intervention [3]. Despite on-going efforts, no effective antiviral drugs are available against the disease and the potential impact of the recently licenced vaccine has yet to be determined. This limits control efforts primarily to vector control [4].

**Fig 1:**
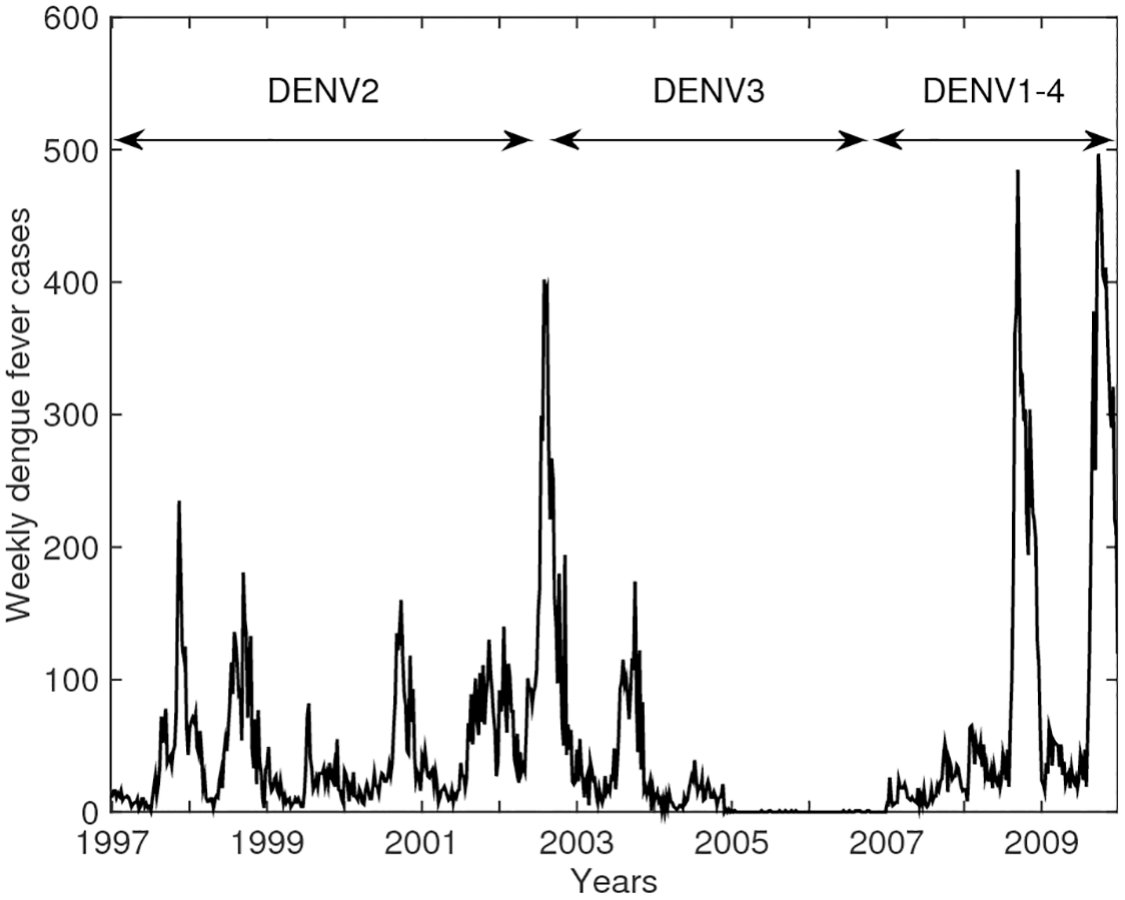
Dengue epidemiology in Trinidad and Tobago. Weekly number of confirmed dengue fever cases with circulating serotypes in Trinidad and Tobago over the period 1997–2009.

Dengue dynamics are characterized by highly seasonal, multi-annual fluctuations, with replacement of serotypes occurring at varying intervals. An example of these patterns arising in a newly emerging dengue setting is illustrated in (Fig 1) [5,6]. This is thought to result from a complex interplay between environmental factors, vector ecology and host-pathogen dynamics [7]. Various hypotheses have been proposed to uncover the main drivers of dengue dynamics and to reveal how such drivers interact among themselves to govern infection and disease patterns in the field. Emphasis has been on unravelling the roles that cross-immunity (CI), cross-enhancement between serotypes, and seasonal variation in the transmission rate, play in capturing the complex dynamics of dengue [8]. Cross-enhancement is believed to be caused by antibody-dependent enhancement (ADE), where heterotypic antibodies facilitate cell entry through the formation of virion-antibody complexes, ultimately leading to increased viral titers upon secondary infection [9,10]. This is thought to result in increased susceptibility to a secondary heterologous infection and, upon these secondary infections, in a more serious form of disease and increased infectiousness. Enhanced disease severity is however believed to have minor impact on the dynamics as the proportion of DHF and DSS cases is substantially small (1% of confirmed cases [11]). By contrast, including sufficiently high levels of enhanced infectiousness or susceptibility (60–130%) in simulation models has been found to induce asynchronous outbreaks of different serotypes [12,13], an outcome which has been indicated to underlie the manifestation of the 3–5 year epidemic cycles observed for dengue dynamics in Thailand [14,15]. Decomposing ADE into both enhanced infectiousness and susceptibility has further been shown to mimic this effect at lower, more realistic values of ADE, while also reducing the magnitude of oscillations to more plausible levels and decreasing the risk of stochastic extinction [15]. Similarly, relaxing the common assumption of complete immunity after two heterologous infections results in asynchronous, multi-annual outbreaks at lower levels of ADE and R_0_ [16]. While most modelling endeavours have assumed serotypes to have identical characteristics, allowing for a small amount of asymmetry in the transmission rate is found to increase serotype persistence in the presence of ADE [17]. Furthermore, the inclusion of short-lived cross-immunity in models was found to be sufficient to reproduce the observed out-of-phase, irregular oscillations and 3-year cycles [18–21]. An alternative hypothesis has been proposed by Lourenço et al., who demonstrated that spatial segregation between human hosts and its vectors can be sufficient to capture the semi-regular dengue patterns observed, even in the absence of immune interactions [22]. By contrast, to mimic the distinct seasonal signature of dengue dynamics, the incorporation of seasonal forcing into the vector population dynamics or transmission rate has been found to be essential [19,22,23].

The above results hint at the complexity of dengue transmission and suggest that multiple mechanisms could underlie disease dynamics in any particular site. A key question in understanding dengue dynamics and control, therefore, is how best to use observed data in order to identify the processes governing the transmission of the disease in a given location. Recently, there has been increasing recognition that for complex systems, such as dengue, model matching to single or a few patterns is not sufficient to narrow down the range of possible explanatory mechanisms [24], and that matching to multiple patterns observed at various scales and hierarchical levels is required for identifying the mechanisms that generate such patterns, and hence are likely to be key elements of the system’s structure. Tying ecological models to multiple system patterns concurrently may also aid in detecting the right level of complexity and improve the predictive ability of such models for replicating local dynamics [24]. Methods such as Pattern Oriented Modelling (POM) allow for such a multi-scope approach by facilitating the design, selection, and calibration of models of complex systems [25–30].

This study applied a POM approach to modelling global dengue infection data in order to determine whether the above proposed mechanisms related to serotype interactions and seasonal forcing of the transmission rate were able to explain all of the observed dynamical patterns in the field. We further used the modelling results to investigate the vulnerability of dengue to interruption in transmission as a result of vector control, and examined how such vulnerability was related to the identified processes governing disease transmission. We demonstrate that model selection is largely driven by the seasonality of the system, with CI being a preferred mechanism in the case of low, and ADE in the case of highly seasonal transmission regimes. At similar levels of transmission rate, resistance to control efforts was found to increase in dengue systems with CI. The results highlight the utility of the POM approach for detecting and fitting of appropriately structured disease transmission models based on observed data. In addition, they also reveal challenges in structural and parameter identifiability that would remain unnoticed when guided by individuals patterns used in isolation.

## Methods

### The patterns in the reported dengue case data

Five characteristic dengue patterns were used to filter out unrealistic model structures and reduce parameter uncertainty. The patterns were selected to reflect the breadth of characteristics used in single pattern matching approaches [12,15,16,18,22], include strong and weak patterns that are common across endemic regions and those which are relatively stable over time and encompass different levels of organization [24]. The patterns (*i*.*e*. mean duration between peaks, multi-annual fluctuations, frequent replacement of one circulating serotype by another, serotype co-dominance and asynchronous serotype cycling) were derived from literature describing dengue case data and serotype epidemiology from different endemic regions across the world [5,6,31–42]. The observed patterns are described in Table 1.

**Table 1:** Characteristics for pattern-oriented modelling.

Characteristics	Range in the literature	Range for analysis		Source
		Lower limit	Upper limit	
Mean inter-peak period	1.4–1.6	1	1.8	[6,34,36–39,41,42]
Multi-annual signals	2–6 years	2 years	6 years	[6,31–35,40]
Duration of serotype replacement	1–6 years	1 years	6 years	[5,6,22,33,36,37,39–41]
Intensity single serotype emergence	Both multi and single- serotype prevalence	0.01	0.99	[5,6,22,33,36–39,41]
Phase-locking	Incomplete	-	-	[5,6,22,33,36,37,39–41]

### The model

**Fig 2:**
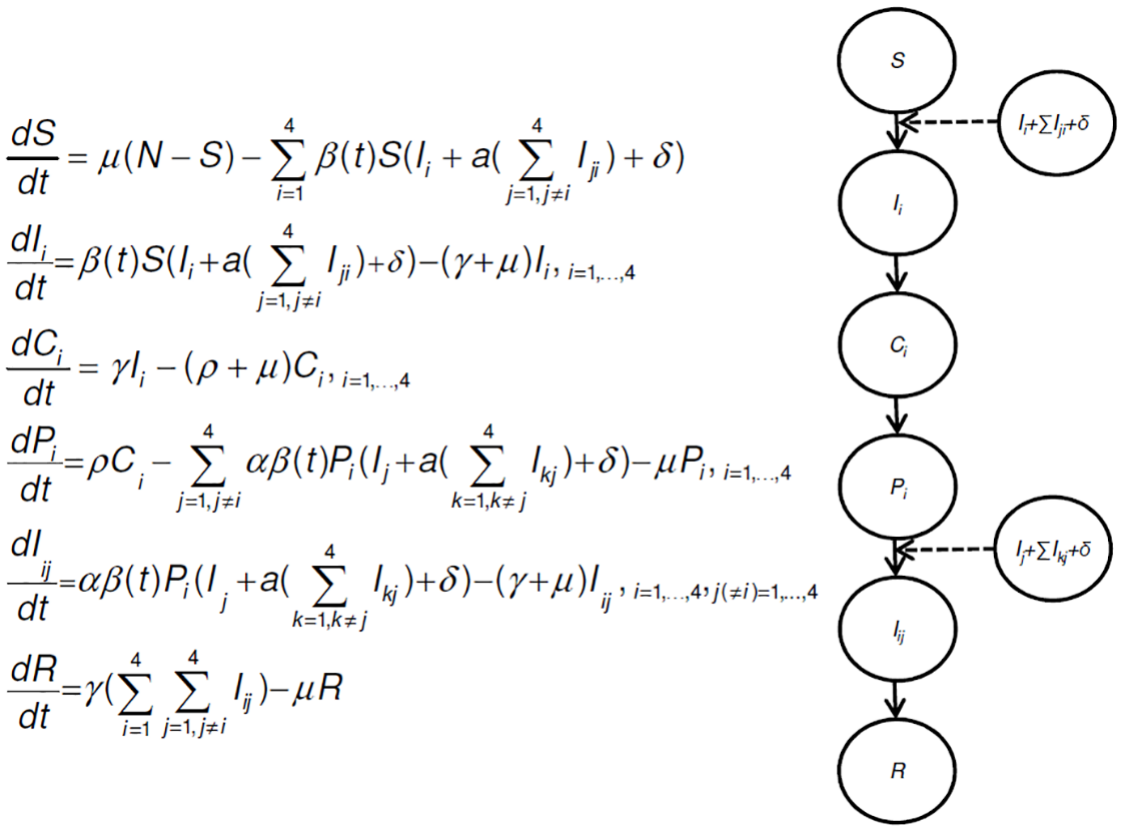
System of differential equations and flow diagram of multi-serotype model. The circles represent the infection related states: susceptible (*S*), infectious (*I*), cross-immune (*C*), partially susceptible (*P*) and recovered (*R*), solid arrows depict the transition from one state to another and the dashed arrows indicate transmission. Parameters are described in Table 3. Simulations are based on a four serotype (DENV1-4) model, where i, j and k denote primary (first subscript) or secondary (second subscript) infection with DENV1-4. The full system consists of 26 compartments. For simplicity, the flowchart for one serotype is shown.

We used a deterministic Susceptible-Infected-Recovered (SIR) modelling framework to describe the circulation of four different dengue serotypes (DENV1-4) in a population [13]. The full system of ordinary differential equations is shown in (Fig 2). The model consists of 26 compartments, each of which represents a fraction of the population. The population size is modelled to be stationary; hence births and deaths occur at an equal rate (*μ*). New-borns are assumed to be immunologically naïve to all serotypes and are born into the class of susceptibles (S). Although the presence of maternal antibodies is shown to affect the risk of infection, the impact on the overall dynamics is believed to be minimal and thus not taken into consideration [43]. Susceptibles become primarily infected by serotype *i* (*I*_*i*_) at rate *βSI*_*i*_ and *α*_*TRANS*_*βSI*_*ji*_ proportional to the number of primarily and secondarily infectious individuals respectively. The parameter *α*_*TRANS*_>1 indicates enhanced transmissibility of secondarily infected individuals. A seasonal change in the transmission rate (*β*(*t*)) is incorporated through a sinusoidal function with a forcing period of one year: *β*(*t*) = *β*_0_(1−*β*_1_ cos(2*πt*)) where *β*_0_ indicates the mean transmission rate and *β*_1_ the strength of seasonal fluctuation and *t* time in years. The transmission rate (*β*(*t*)) is assumed to be equal across serotypes. Individuals remain infectious for a period of 1/*γ*. After recovery from a primary infection, individuals become immune to all serotypes (*C*_*i*_) for a period 1/ρ after which they move to the partially immune stage (*P*_*i*_). The P-class individuals are assumed to experience full immunity against the serotype *i* and enhanced susceptibility (*α*_*SUS*_>1) to all other serotypes. They acquire secondary infection (*I*_*ij*_) at rates *α*_*SUS*_*βP*_*i*_*I*_*j*_ and *α*_*TRANS*_*α*_*SUS*_*βP*_*j*_*I*_*kj*_ proportional to the number of cases respectively primarily and secondarily infectious to a different serotype (with *k*≠*j* and *j*≠*i*). The duration of the infectious period is assumed to be equal upon secondary and primary infection. To account for imported cases and prevent the ODE-models to simulate unrealistically low levels of infections, individuals (susceptible or partially immune) can also acquire infection through an infectious contact with an individual from an external population at rate *βδ*, where *δ* signifies the import rate [23]. As tertiary and quaternary infections are rarely observed [44], we assume that after recovery from a secondary infection, individuals become life-long immune to all serotypes. An adaptive time step fourth and fifth -order Runge-Kutta solver was used with initial conditions for I_1-4_ 1x10^-7^, 2x10^-7^, 3x10^-7^ and 4x10^-7^ and $S=1-\sum_{1-4}^{i}I_{i}$. All other state variables were initialized at zero. The implementation of the model, as well as the analysis of its simulation results were carried out in the Matlab, version 2014b (www.mathworks.com).

### Model hypotheses

In this analysis we assume the following hypotheses (see Table 2). H1: The most parsimonious hypothesis is represented by the base-model with neither ADE (*α*_*SUS*_ = 1 and *α*_*TRANS*_ = 1) nor CI (individuals upon recovery from primary infection go straight to the P-class). H2: The base-model with CI. H3: The base-model with enhanced susceptibility, further referred to as ADE (*α*_*SUS*_>1 and *α*_*TRANS*_ = 1). H4: H3 with CI. H5: The base-model with both enhanced susceptibility and transmissibility (*i*.*e*. ADEx2 with *α*_*SUS*_>1 and *α*_*TRANS*_>1) but no CI. H6: H5 with CI. In all models, an annual seasonal forcing in the transmission rate is assumed.

**Table 2:** Model hypotheses.

	Model	Seasonality	Cross-Immunity	Enhanced susceptibility	Enhanced transmissibility
1	Base	X			
2	CI	X	X		
3	ADE	X		X	
4	ADE+CI	X	X	X	
5	ADEx2	X		X	X
6	ADEx2 + CI	X	X	X	X

Models are built as described in Fig 2. In the absence of cross-immunity, individuals are assumed to move straight from the infectious state (*I*) to the partially susceptible state (*P*). In the absence of enhanced susceptibility and enhanced transmissibility α_SUS_ and α_TRANS_ respectively, are set equal to 1.

### Defining dengue characteristics in simulated data

The variables that we estimated from the simulated data to contrast the dynamics of each model against the characteristics of dengue dynamics are: 1) Mean inter-peak period; 2) Presence of a multi-annual signal; 3) Duration of serotype replacement; 4) Intensity of single-serotype emergence; and 5) Serotype phase-locking.

The *mean inter-peak period* (MIPP) is defined as:$MIPP=\frac{Y}{N}$, where *Y* is the number of years analysed and *N* the number of peaks occurring during that period. To ensure comparability of the simulated estimates with reported observations on the inter-epidemic period, peaks were defined to have a minimum proportion of infectious people of 1/4000. To assess the presence of significant *multi-annual signals* in addition to the near yearly MIPP, a spectral density approach was used. To reduce the confounding effect of very low amplitude fluctuations, the time series were smoothed using a moving average filter. The power spectral density of the smoothed time series was assessed with the Welch’s overlapped segment averaging estimator [45]. To evaluate the significance of the periodic signals, the signals were compared to the null-continuum. The null-continuum is a greatly smoothed version of the raw periodogram, encapsulating the underlying shape of the distribution of variance over frequency [46]. A signal was assessed to be significant if the lower bound of the 90% confidence interval of the raw periodogram exceeded the null continuum [46]. The *duration of serotype replacement* is defined as the mean number of years before a dominant serotype during a peak is replaced by another serotype in a subsequent peak. The intensity of single serotype emergence (*ε*) was defined as by Recker et al. [47]:$\varepsilon =\frac{1}{N}\sum_{i}^{N}\frac{\gamma_{\max}^{i}-\gamma_{sub}^{i}}{\gamma_{\max}^{i}},$ where *N* defines the number of peaks occurring during the analysed number of years,$\gamma_{\max}^{i}$ the prevalence of the dominant serotype and $\gamma_{sub}^{i}$ the prevalence of the serotype with the second-highest peak. Model runs with either complete co-dominance (*ε*<0.01) (*i*.*e*. there are multiple serotypes present at any point in time) or complete single serotype dominance (*ε*>0.99) were omitted. Lastly, *serotype phase-locking* here is defined as the perfect synchronization of serotypes and is detected by comparing the MIPP of serotype *i* to the aggregated MIPP. Simulations in which *MIPP* = *MIPP*_*i*_ are discarded based on the presence of perfect phase-locking.

### Data-model pattern matching

**Fig 3:**
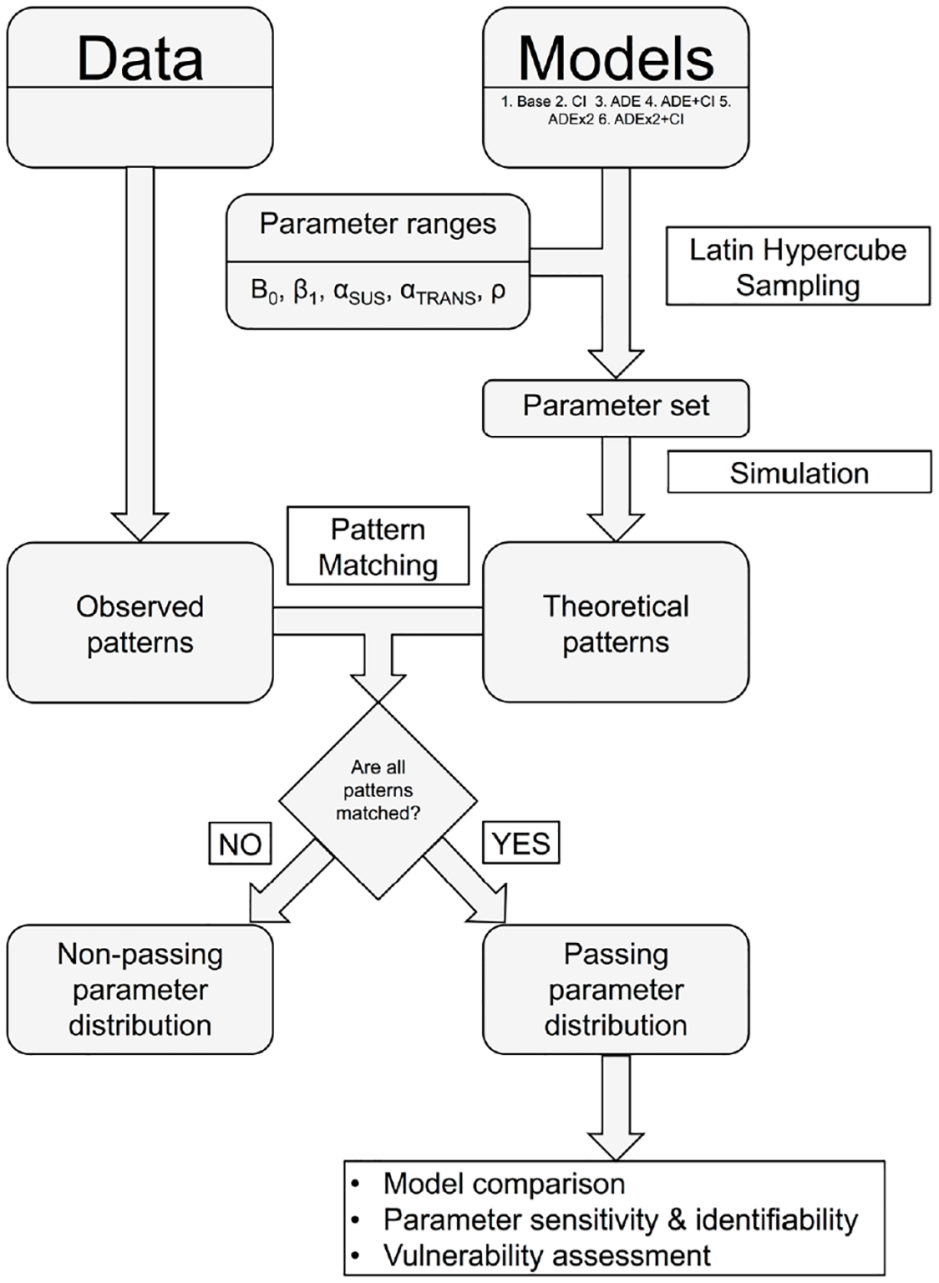
Flow chart of Pattern Oriented Modelling approach. A set of 6 alternative models are identified and compared with respect to their ability to replicate patterns observed in dengue case data. Each model is run for a set of 5,000 different parameter combinations, sampled from plausible parameter ranges using Latin hypercube sampling. The resulting patterns from each simulation are compared to the observed patterns. The parameter sets that match all 5 patterns of interest are assembled into the passing parameter set, which forms the input for model comparison and the examination of model behaviour.

To determine which of the hypotheses or models capture the observed dengue dynamics and at which parameter values, we used a pattern oriented modelling approach (Fig 3) [25–28]. Model performance was assessed based on the extent to which a model captured all the 5 characteristics of dengue simultaneously, as defined above (Table 1). Models were assessed using the following steps. First, Latin hypercube sampling [48] was employed to select a sample of Ω (= 5,000) parameter vectors from a conjoint parameter distribution, encompassing the transmission rate (β_0_), the level of seasonal forcing or seasonality (β_1_) and, depending on the model, a combination of enhanced susceptibility (*α*_*SUS*_), enhanced transmissibility (*α*_*TRANS*_) and the rate of loss of CI (*ρ*) (Table 3). Uncertainty in the values of these parameters was addressed by assigning uniform distributions from their ranges deemed realistic according to literature (Table 3). The resulting ensemble of models (Model 1–6 with Ω parameter vectors) was run for 1400 years. The model outputs for the last 400 years were considered to determine whether the model mimicked all five dengue characteristics (a model is assumed to match a characteristic if the simulated response falls within the range of that characteristic pattern given in Table 1). The resulting set of passing (good) parameters G (where G ⊂ Ω) was retained as a multivariate distribution for further analysis.

**Table 3:** Model parameters.

Symbol	Description	Value	Range	Source
β_0_	mean transmission rate, year^-1^	400	100–400	[13,18]
β_1_	seasonal forcing	0.05	0–0.35	[20,23]
*μ*	host life expectancy, year^-1^	1/70	fixed	[13]
*γ*	recovery rate, year^-1^	100	fixed	[13,49]
*ρ*	1/ duration of cross-immunity, year	2	1/3–3	[23]
*α*_*TRANS*_	infectiousness enhancement	>1	1–2.4	[15]
*α*_*SUS*_	susceptibility enhancement	>1	1–2.4	[15]
*δ*	import rate	1e-10	fixed	[20,23,50,51]

Parameter values used in the model simulations, where Value indicates the best estimate from literature and Range depicts the boundaries of the uniform prior from which is sampled for the POM-approach.

### Sensitivity analysis

To assess the impact of simplifying model assumptions on pattern-matching, we repeated the POM exercise for two distinct scenarios. One, we allowed for transmission rates to be uneven between serotypes (the asymmetric model). More specifically, serotype-specific transmission rates were drawn from a normal distribution with standard deviation 0.15 [17]. Two, we used a model variant that allows for four heterologous infections prior to acquiring complete immunity (the 4-infection model, equations are provided in S1 Text [52]).

### Parameter sensitivity and identifiability

We used logistic regression to assess the sensitivity of pattern-matching (binary response variable) to the parameters (independent variables). We normalised the independent variables on a 0 to 1 scale to obtain comparable regression coefficients: coefficients larger than|3| indicate strong sensitivity while parameters with small coefficients (<<|1|) have little impact on the model matching the patterns [53]. Two-way interactions were included in the construction of the logistic regression models: logit(*p*) = *b*_0_+*b*_1_*β*_0_+*b*_2_*β*_1_+*b*_3_*α*_*SUS*_+*b*_4_*α*_*TRANS*_+*b*_5_*ρ*+interactions, with *p* being the probability of a pattern-match, *b*_0_ the intercept and *b*_1-n_ the regression coefficients.

Additionally, the identifiability of each of the parameters was examined using a principal component analysis (PCA) [54,55]. The identifiability of a parameter is a function of dependence, prior uncertainty and the model’s sensitivity to the parameter and defines how well one can estimate a parameter. We assessed the parameter identifiability for the full model (ADEx2+CI), using its passing distribution (G). First, the variance-covariance matrix(Σ)was constructed from the log-transformed G. Next, the principal components (PCs) were derived from Σ. The PCs of Σ define the 5-dimensional ellipsoid that approximates the population of passing parameter values. The eigenvalues (*λ*_*i*_) denote the respective radii and the eigenvectors representing how much each parameter contributes to the direction of each radius. As such, *λ*_*i*_ gives an indication of the variance explained by the *i*^*th*^ PC. The overall variance of all PCs was defined as $\sum_{i=1}^{5}\lambda_{i}=trace\left(\Sigma \right)$, thus the proportion of the total variation in G that was explained by the *i*^th^ PC is was estimated by:$\frac{\lambda }{trace(\sum )}.$ We interpret these results as follows: A smaller *λ*_*i*_ indicates that the model is more sensitive to changes in the direction described by the *i*^th^ component, whereas a larger *λ*_*i*_ signifies that the model is less sensitive to changes in the direction of the component. Parameters contributing most to a large *λ*_*i*_ are responsible for a big portion of the variation in the parameter space and are thus considered less identifiable.

### Vulnerability to disruption in dengue transmission

We examined the vulnerability of the models to sudden reductions in the transmission rate that may be brought about by vector control. The models were run for all parameter sets in G for a burn-in period of 1000 years after which the system was perturbed by a reduction in the transmission rate (*i*.*e*. β_0_ is reduced by 90%) for a control period of *w* weeks per year. We varied *w* from 1 week to 52 consecutive weeks, starting at the valley of the sinusoidal function, which mimics the onset of the rainy season. After the control period of *w* weeks, β_0_ returns to its original value. These control runs were performed for 30 years after the burn-in period. The intervention of *w* weeks was assumed to be successful if no more than one peak occurred over the time-course of the model simulation. We assessed the probability of control for model *i*, where *i* represents 1 to 6, by calculating the proportion $\left(P_{w_{i}}\right)$ of G_*i*_ presenting successful control as a function of the number of weeks the transmission was disrupted. Here, $P_{w_{i}}=\frac{N_{w_{i}}}{G_{i}}$ with $N_{w_{i}}$ being the number of parameter vectors out of G_i_ that showed successful control for model *i* given *w* weeks of interruption in transmission. A composite average (*P*_*w*_) for each control period *w* was derived by weighing the individual probability values of the models by the sizes of their passing parameter distributions (G_*i*_), such that:$P_{w}=\sum_{i=1}^{6}\frac{N_{w_{i}}}{G_{i}}$.

Lastly, we estimated the values of the basic reproduction rate (R_0_) for each of the parameter vectors in G to assess the relation between transmission potential and the models’ vulnerability. The R_0_ of the model was derived using the next generation method [56–58] (Proof provided in S2 Text) and is defined as: $R_{0}=\frac{\beta_{0}}{\gamma +\mu }$, where β_0_ defines the transmission rate, 1/*γ* the duration of the infectious period and 1/*μ* the average life expectancy of the human host [59].

## Results

### Model performance

#### 2-infection models

We compared the ability of six 2-infection models to reproduce the main characteristics of dengue epidemiology listed in Table 1. Table 4 shows the proportions of parameter sets for which the models were able to capture the dengue dynamics by reproducing the five characteristics, either all simultaneously (values in bold) or each individually. Each of the six models investigated in this study was capable of simultaneously reproducing the five patterns of dengue dynamics, albeit at different proportions of the parameter space. The percentage indicates how robustly a model could replicate the patterns across the parameter space. While each pattern, independent of the others, could be reproduced at a relatively high probability, the simultaneous reproduction of all five patterns was found to occur rarely. In general, one would expect models with increasing complexity to perform better than simpler models. Indeed, the full model performed best overall (10.98%). However, here, the base-model was found to perform nearly as good as the second best model (*i*.*e*. the ADE+CI model); the respective overall proportions were similar in magnitude (5.54% versus 5.76%). Both the CI- and ADE-only models performed poorly, with overall proportions of 1.16% and 2.02%, respectively. The model with the decomposed ADEs approximately performed twice as well as either of these two models.

**Table 4:** Model performance.

	2-infection symmetric model	2-infection asymmetric model	4-infection symmetric model
**Base-model**	**5.54**	**4.34**	**0.06**
Mean inter-peak period	74.2	65.8	95.2
Multi-annual signal	34.6	46.6	59.2
Duration of serotype replacement	49.3	43.2	12.5
Single serotype emergence	34.6	92.3	57.3
Absence of phase-locking	10.7	14.3	16.1
**CI**	**1.10**	**1.20**	**21.9**
Mean inter-peak period	27.9	22.3	63.4
Multi-annual signal	91.7	90.8	87.3
Duration of serotype replacement	87.6	90.6	70.2
Single serotype emergence	87.0	96.8	95.8
Absence of phase-locking	34.0	40.4	76.4
**ADE**	**1.88**	**7.04**	**1.94**
Mean inter-peak period	88.0	71.9	78.2
Multi-annual signal	63.7	64.2	64.6
Duration of serotype replacement	23.1	42.4	24.1
Single serotype emergence	54.8	96.9	84.7
Absence of phase-locking	18.2	33.2	74.7
**ADE+CI**	**5.76**	**5.70**	**12.54**
Mean inter-peak period	41.7	33.2	78.1
Multi-annual signal	91.8	88.8	83.2
Duration of serotype replacement	81.6	86.0	38.5
Single serotype emergence	86.5	97.9	98.8
Absence of phase-locking	42.0	52.6	88.7
**ADEx2**	**3.4**	**7.06**	**0.96**
Mean inter-peak period	47.6	38.9	28.9
Multi-annual signal	73.3	71.4	66.0
Duration of serotype replacement	45.2	62.2	53.8
Single serotype emergence	79.2	99.1	94.2
Absence of phase-locking	72.4	86.1	94.0
**ADEx2+CI**	**10.98**	**9.78**	**4.82**
Mean inter-peak period	50.9	50.2	76.1
Multi-annual signal	84.7	79.7	77.2
Duration of serotype replacement	62.5	62.6	20.0
Single serotype emergence	89.8	98.6	98.9
Absence of phase-locking	91.2	94.6	97.5

Percentage of runs (n = 5,000) that meets the characteristics of dengue dynamics for each model structure. In the 2-infection symmetric model, heterologous immunity is assumed after a second infection and serotypes are assumed to have equal transmission rates. In the 2-infection asymmetric model, the four serotypes differ in transmission rates. In the 4-infection symmetric model, no heterologous immunity is assumed until one has recovered from all four serotypes. ADE = antigen dependent enhancement and CI = cross-immunity.

The performance of each model can also be examined by their ability to reproduce each characteristic separately. In this case, the base-model generally performed worse than the other models, yet it appeared to be equally proficient at simultaneous reproducing all characteristics or patterns as the ADE+CI-model, a more complex model than the base-model. While the MIPP is best captured by the base- and ADE-model (Table 4), all other characteristics demonstrate preference to the models that include CI. The model’s proficiency to reproduce the multi-annual signal however interferes with its ability to capture the seasonal signature in the MIPP (Table 4). As such, the POM methodology appears to penalize for overly specialized model hypotheses.

Both the base-model and ADE-model are hampered in their performance by large regions of phase-locking (S1aEA and S1aEC Fig) and to a lower extent, complete single serotype dominance (S1aDA and S1aDC Fig). The parameter space in which phase-locking occurs is largely reduced by the addition of decomposed ADE as well as CI, which both induce irregular, asynchronous serotype circulation (S1aED and S1aEE Fig).

#### Asymmetric 2-infection models

Relaxing the assumption of symmetry in transmission rates does not affect the level of overall fit of the base-model or any of the models with CI. However, models with ADE or decomposed ADE performed better upon the inclusion of asymmetry. Across all models, the parameter space at which complete serotype co-dominance occurred was reduced by the inclusion of asymmetric transmission rates. This co-dominance seemed to be a strong constraint on the ADE and ADEx2 models in the 2-infection case and is the reason for the markedly improved fit in the asymmetric case.

#### Symmetric 4-infection models

The impact of the relaxing the assumptions of full immunity after the second heterologous infection is substantial. The simple CI-model performed far better than any other models, with a 10-fold increased performance relative to its 2-infection counterpart. In the 4-infection case, the performance of the full model was about twice better than the 2-infection case. This is largely due to reduced phase-locking in the 4-infection case [16]. The phase-locking was the foremost restricting factor of the CI-model in the 2-infection case. The base-model in this case, however, showed a markedly reduced performance as a result of shortened time required for serotype replacement. This indicates that the permanent heterologous immunity only after two infections was the driver of the serotype interactions sufficient to result in desynchronized oscillations in the 2-infection base-model. The few fits (0.06% of 5000, see Table 4) of the base-model occur because of an additional implicit serotype interaction. Since no more than one infection is assumed to occur concurrently, this introduces short cross-immunity that lasts for the infectious period. Indeed, when we allowed for more than one infection in the 4-infection base-model, the out-of-sync oscillations disappear completely (S6 Fig).

### Model calibration and selection

**Fig 4:**
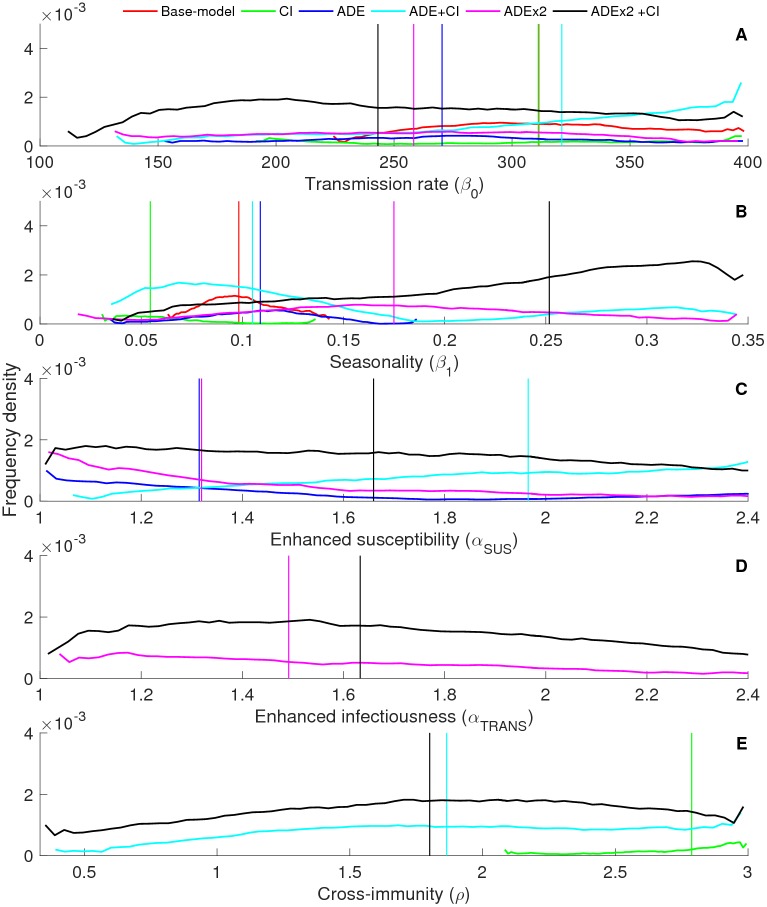
Model parameter distributions. Parameter distributions for passing parameter sets (G) for different model hypotheses (with ADE = antibody dependent enhancement, CI = cross-immunity) for (A) the transmission rate (β_0_), (B) seasonality (β_1_), (C) enhanced susceptibility (α_SUS_), (D) enhanced infectiousness (α_TRANS_), and (E) 1/duration of cross-immunity (ρ). The vertical lines depict the median values for each distribution with the colours indicating the corresponding model hypothesis.

Fig 4 demonstrates the accepted parameter distributions (G) for the 2-infection models. While some parameters demonstrate broad distributions indicating limited uniqueness and abundant parameter interactions, others show clear preferential values and ranges that are sensitive to the structural components of the model. Overall it appears, as can be expected, that the more complex models fit the patterns at a wider parameter range.

Fig 4A shows that models with CI selected for relatively higher transmission levels relative to models with ADE only. For low transmission levels, the full model outcompeted all the other models, indicating that more complex models may be necessary to fit dengue dynamics at lower values of R_0_. These results are insensitive to the assumption of low levels of asymmetry in transmission rates (S2aA Fig). In contrast to this, the 4-infection models display similar fits at lower transmission levels (S2bA Fig).

Seasonality appeared to be the most prominent driver of model fit and selection in the 2-infection model (Fig 4B). Models with CI showed a marked shift towards lower seasonal forcing relative to the base-model. In fact, at low seasonality (β_1_<0.06) there is a strong preference for the inclusion of CI, as is especially notable from the elevated density levels of the ADE+CI and ADEx2+CI models. At high seasonality (β_1_>0.17) only the more complex models provided an adequate fit. At intermediate levels of seasonality (β_1_: 0.1–0.15) multiple models were equally proficient at replicating the dynamics, indicating a region of large model uncertainty. The model’s structural sensitivity to seasonality persisted when asymmetry in transmission rates was assumed (S2aB Fig). However, when we allowed for tertiary and quaternary infections, the medians and shapes of the passing parameter distributions for β_1_ were similar across the models (S2bB Fig).

The addition of CI to models with ADE results in higher levels of *α*_*SUS*_ (Fig 4C), yet had minor impact on the median levels of *α*_*TRANS*_ (Fig 4D). While previous publications suggested reduced estimates of *α*_*SUS*_ and *α*_*TRANS*_ upon the inclusion of decomposed ADE, analysis of the 2-infection model does not support this observation [15]. We did, however, observe this pattern in the 4-infection and asymmetric 2-infection model (S2aD and S2bD Fig).

The inclusion of ADE to the models with CI profoundly affects the estimated duration of cross-immunity by allowing for the selection of a much wider range of ρ (Fig 4E). Whereas the CI-model by itself only captures the characteristics at durations of cross-immunity shorter than half a year, the inclusion of ADE allows for cross-immune periods of up to 2 years, which is in line with the previous estimates [21]. Interestingly, in the case of 4-infection, the CI-only model performed well for a wider range of durations of cross-immunity, including estimates from Reich et al. [21].

### The role of seasonality and cross-immunity

Exploring the behaviour of the models in terms of MIPP and duration of serotype replacement (Table 4) reveals as to why there are differences in model fits across the range of seasonal forcing (S1aAA–S1aAF and S1aCA–S1aCF Fig). Increased levels of seasonal forcing are associated with longer MIPP. Temporary CI introduces a lag before a secondary infection can be acquired and thus generates a necessary build-up time period during which susceptible individuals accumulate in sufficient number to fuel the next outbreak. Thus, while an increase in seasonal forcing is characterized by longer inter-epidemic periods, at similar levels of seasonal forcing, the models with CI demonstrate a longer MIPP than the models without CI (S1aAA–S1aAF Fig). This allows the CI-only models capture the characteristic MIPP at lower seasonal levels than the models with just ADE. At higher levels of seasonal forcing, CI contributes to MIPPs that are longer than are characteristic to dengue. This effect is less pronounced in the 4-infection models. The overall immune population is smaller in the 4-infection models and therefore of less influence on the frequency of outbreaks. The same can be observed for the duration of serotype replacement (S1aCA–S1aCF Fig). In contrast to CI, the inclusion of ADE to the model results in shorter cycles, thus successful fits are observed at higher levels of seasonal forcing (S1aAA–S1aAF Fig).

Lastly, we observe a prominent impact of seasonal forcing on the occurrence of phase-locking. S1aEA–S1aEF Fig demonstrate a threshold-like value of β_1_ above which the system is forced into synchronized serotype dynamics. This threshold is relatively stable across the simple model structures (see also Fig 4B) and unaffected by the value of R_0_. Only the addition of decomposed ADE disrupts this behaviour, thereby being a possible driver of irregular serotype behaviour at higher seasonal regions. These phase-locking thresholds are stable to some level of asymmetry in transmission rates (S1bEA–S1bEF Fig), however they completely vanish in the case of 4-infection models (S1cEA–S1cEF Fig).

### Parameter sensitivity and identifiability

The logistic regression coefficients for the full-model given in Table 5 illustrate the differential roles each of the parameters play in explaining the dengue characteristics. β_0_ is found to be an important driver of the multi-annual signal. And in conjunction with β_1_ and *α*_*TRANS*_, it is the dominant factor for the absence of phase-locking. As can be expected, β_1_ is the main driver for reproducing a seasonal signature. The parameter for CI (ρ) interacts with β_1_ in reproducing this pattern and is thus also an important determining factor in fitting the MIPP. The R^2^-values for each of the regression models illustrate that the separate parameter values provide reasonable information about whether a characteristic is met or not. However, when assessing the simultaneous fit, the predictive power of the parameters is negotiated by interactions between the parameters and the separate characteristics. In particular the interactions between β_1_ and *ρ* govern simultaneous fitting (S3a Fig). These interactions are conserved when fitting the asymmetric 2-infection and symmetric 4-infection model (S3b and S3c Fig).

**Table 5:** Sensitivity analysis of model fit full model.

Pattern	R^2^	P-value	Coefficients					
			intercept	β_0_	β_1_	*α*_*SUS*_	*α*_*TRANS*_	*ρ*
**Mean inter-peak period**	0.49	<0.005	**-9.84**	0.57^1^	**15.3**	1.37^2^	1.00^3^	**5.99**
**Multi-annual signal**	0.21	<0.005	**4.43**	**3.08**	-2.49	-2.33	**-3.25**	-2.35
**Duration of serotype replacement**	0.47	<0.005	**4.43**	2.09	**-4.96**	0.91^4^	-0.27^5^	0.06^6^
**Intensity single serotype emergence**	0.08	<0.005	0.75^7^	0.39^8^	1.09^9^	0.64^10^	1.33^11^	0.51
**Phase-locking**	0.52	<0.005	-0.35^12^	**6.27**	**-3.28**	2.10^13^	**4.61**	2.84
**Simultaneous fit**	0.07	<0.005	**-7.88**	2.93	**4.23**	**4.06**	2.69	**5.94**

Logistic regression model coefficients with pattern-match as binary response variables and the parameters (scaled 0–1) as independent variables. Two-way interactions are taken into account (coefficients in S1 Table). Bold are high coefficient values (>|3|). Coefficients are significant (p<0.005) unless stated otherwise:

^1^p = 0.04,

^2^p = 0.03,

^3^p = 0.12,

^4^p = 0.15,

^5^p = 0.67,

^6^p = 0.93,

^7^p = 0.1,

^8^p = 0.52,

^9^p = 0.08,

^10^p = 0.28,

^11^p = 0.40,

^12^p = 0.58,

^13^p = 0.02

**Fig 5:**
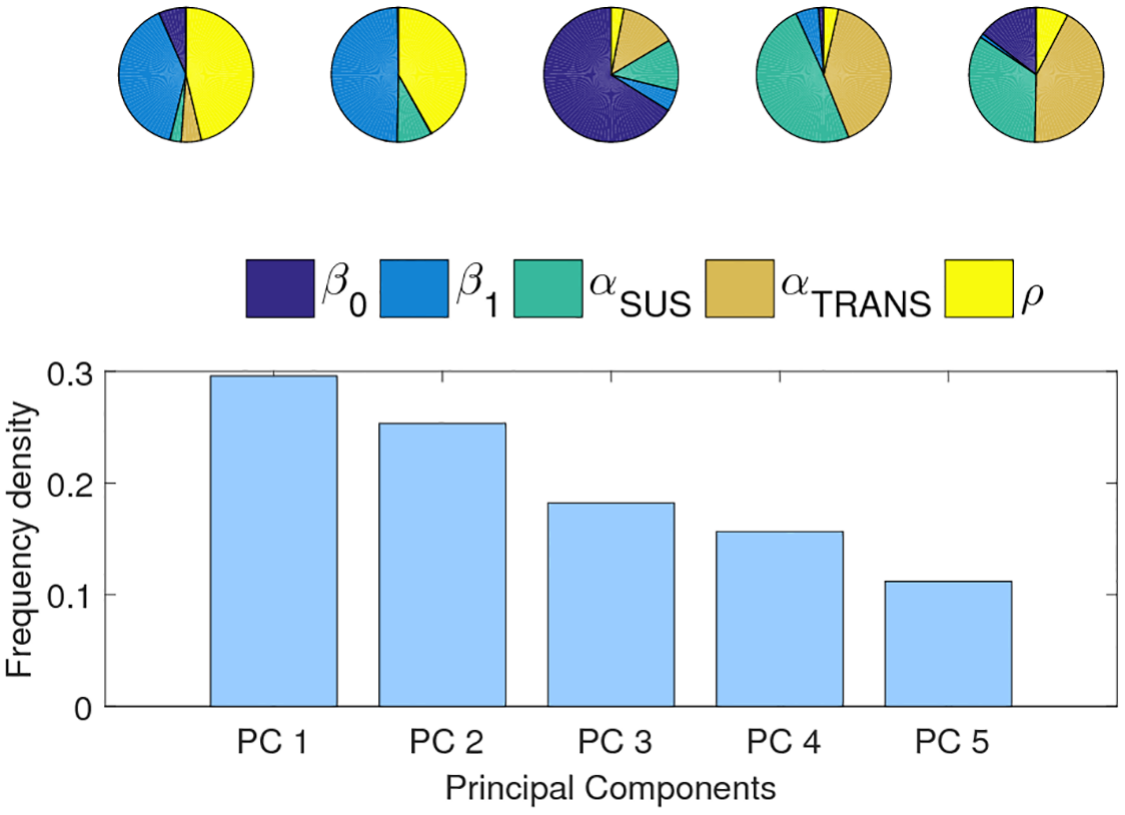
Principal component analysis. Principal component analysis of passing parameter space (G) of the full model (ADEx2+CI). The first component explains 30% of the total variance, the second 25%, the third 18% and fourth 16% and the 5^th^ 11%. The pie charts show the contribution of the parameters to each component. β_1_ and *ρ* dominate the first component, indicating reduced identifiability. β_0_, *α*_*SUS*_ and *α*_*TRANS*_ dominates the fifth component and thus contribute most to the stiffest (*i*.*e*. most sensitive direction in the parameter space).

Strong, multi-level parameter interactions typically result in limited parameter identifiability. Indeed, the PCA reveals that, in particular the estimates for β_1_ and *ρ* are found to be little constrained by the characteristic patterns (Fig 5). The parameters β_1_ and *ρ* dominate the first two components, which explain the largest portion of the total variance in the passing parameter space (G_full_) (55%). While this observed lack of uniqueness may result from the limited influence the parameters have on replicating the dynamics and the substantial width of the criteria, complex interactions between patterns and parameters can also underlie this phenomenon. Indeed, as observed earlier, β_1_ and *ρ* are correlated with each other as well with other model parameters, which substantially impedes parameterization efforts (S3a Fig). Parameters β_0_, *α*_*SUS*_ and *α*_*TRANS*_ contribute equally to the smallest component, indicating that these are more constrained by the examined characteristics and the level of uncertainty and are less affected by dependence to other parameters (Fig 5). Allowing for asymmetry in transmission or tertiary and quaternary infections reduces the contribution of seasonality to the first component, leaving the duration of cross-immunity as the most important factor in explaining the variance in the passing parameter distributions (S5a and S5b Fig).

### Vulnerability to disruption in dengue transmission

**Fig 6:**
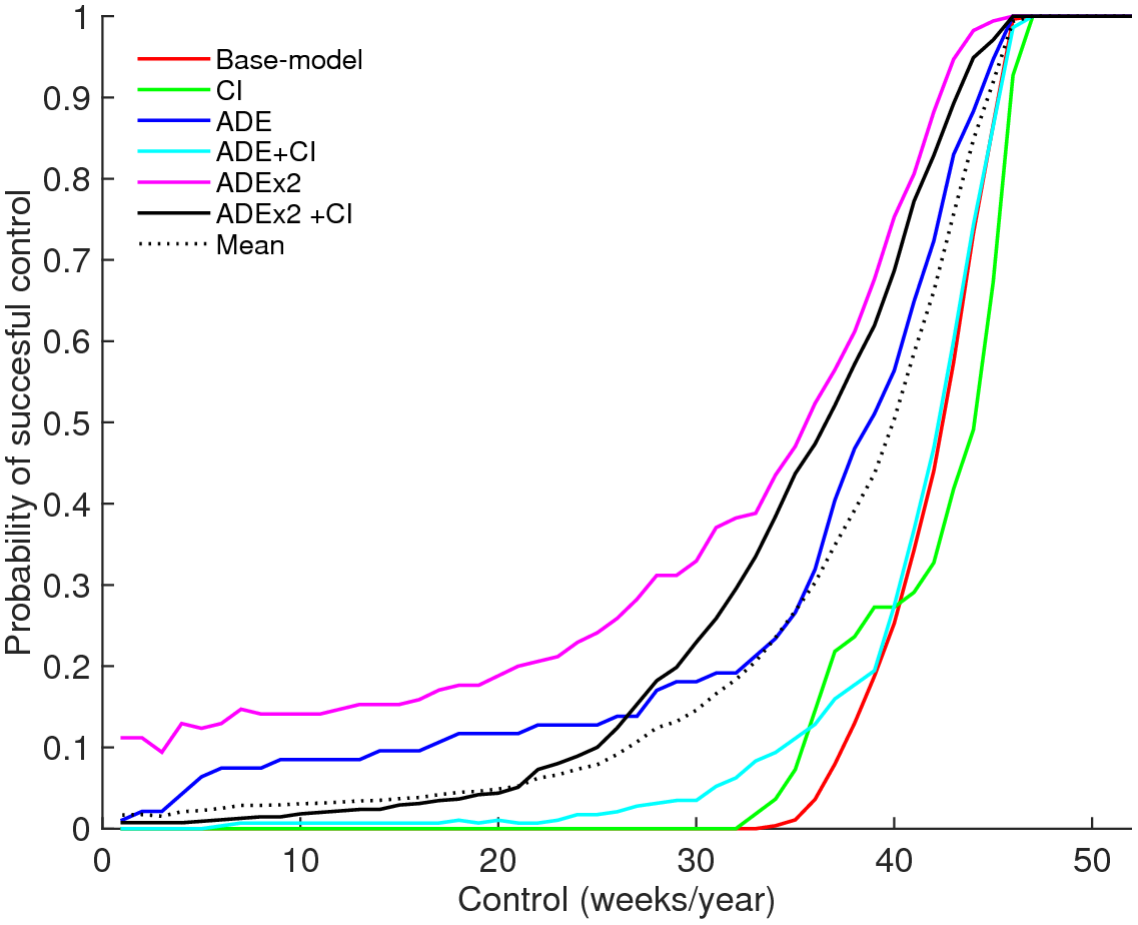
Overall vulnerability to control. Probability of successful control (a maximum of 1 outbreak during 30 years) given the duration (weeks/year) of consecutive control (temporary reduction of transmission: β_0_(1–90%) for different model hypotheses (with ADE = antibody dependent enhancement, CI = cross-immunity). The probability is defined as the proportion of the passing parameter sets (G_i_) that reach successful control. Here *i* refers to the six models, shown by the individual keys. The dotted line shows the mean probability across all models.

Fig 6 depicts the probability of achieving successful control (≤ 1outbreak in 30 years) as a function of *w* weeks of reduced transmission (e.g. due to implementation of vector control). The duration of control required to reach a desired probability of successful control can be used to quantify the level of resistance or vulnerability of a dynamical transmission system.

The inclusion of ADE or ADEx2 reduces the resistance of the model to perturbations (dark blue and pink lines), provided no CI is assumed (Fig 6). Including CI to the model offsets this effect and demonstrates a resistance profile similar to the base-model at longer control efforts, yet shows larger vulnerability at shorter durations of control. The exception is the full-model, which converges with the ADE-model at longer control durations.

**Fig 7:**
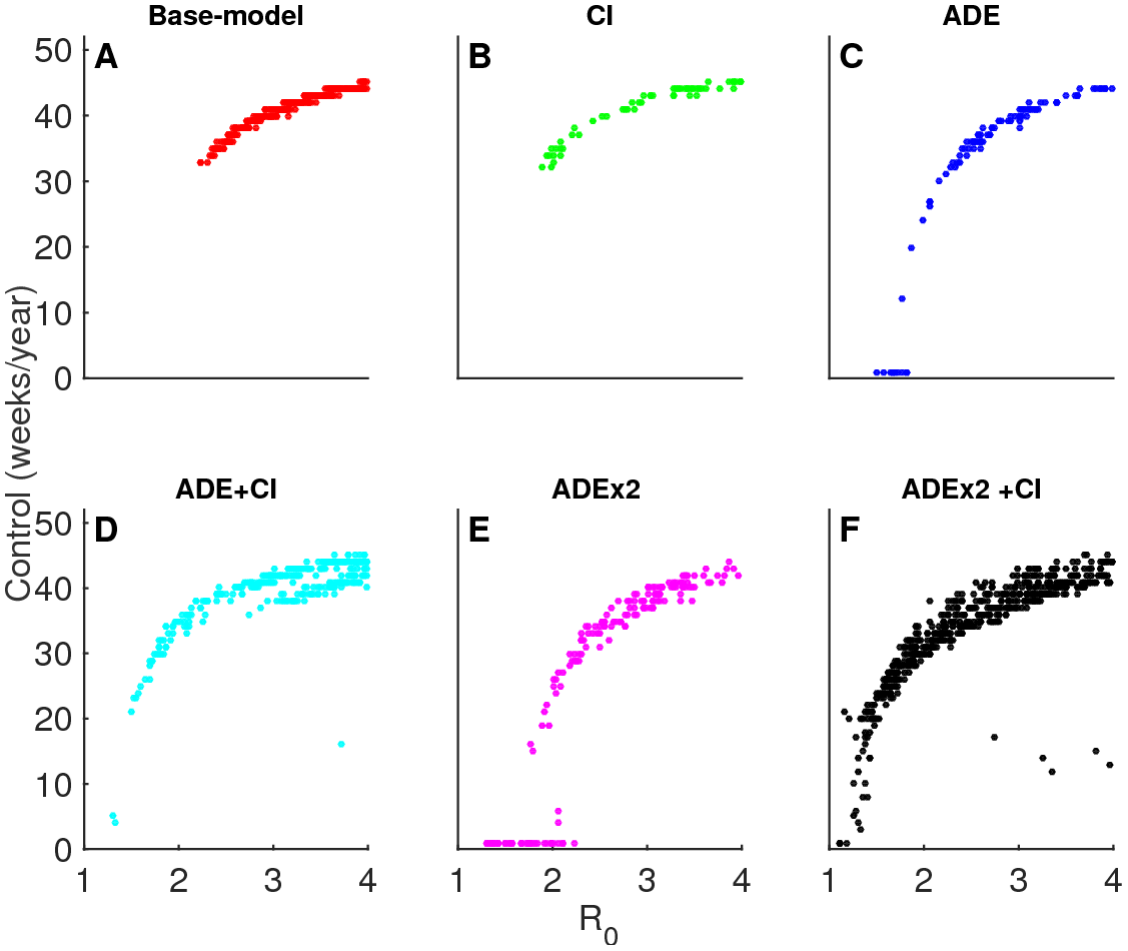
Vulnerability to control as a function of R_0_. Required duration (weeks/year) for achieving successful control is shown with respect to the basic reproduction number R_0_ (= β_0_/(*γ*+*μ*)) for the different model hypotheses: are base (A), CI (B), ADE (C), ADE+CI (D), ADEx2 (E), and ADEx2+CI (F), with ADE = antibody dependent enhancement, CI = cross-immunity.

The large resistance to control in the base-model is a consequence of the high values of R_0_ required for this model to meet the criteria (R_0_>2.2) (Fig 7A). At those levels of R_0_ the ADE-model demonstrates higher vulnerability to control as a result of decreased persistence (Fig 7C). The enhanced vulnerability of the ADE-model relative to the base-model as seen in Fig 6 is a consequence of low transmission rates. The inclusion of CI to either model enhances the resistance of the model especially at lower values of R_0_ (Fig 7D). Longer durations of cross-immunity are associated with greater resistance (S7DE Fig), while increased enhancement results in decreased resistance (S7CC and S7DC Fig).

This differential vulnerability is in part due to low infection persistence levels, a typical property of models with ADE only [12,15,23]. The addition of CI counters this effect with and without ADE (Fig 7C, 7D and 7F). This difference in infection persistence between CI and ADE systems, however, diminishes at high levels of seasonal forcing and R_0_. At these high transmission levels, both the models with CI (ADEx2+CI) and without CI (ADEx2) represent extreme fluctuations and long periods of non-persistent dynamics (S4aF and S4aG Fig). Thus, the differential model preference affects predicted control efforts more substantially in lower than higher seasonal scenarios.

## Discussion

We used a pattern-oriented modelling approach to test a range of multi-serotype models and parameter values for their ability to simultaneously replicate multiple dengue fever patterns derived from literature (Table 1) and case data from Trinidad and Tobago (Fig 1). Despite using such a multiple-pattern data fitting approach, we show that all the investigated model structures were effective at fitting each of the characteristic dengue patterns across some part of the model parameter space, suggesting the occurrence of equifinality, *i*.*e*. that observed infection patterns can be reproduced by more than one mechanism or combinations of mechanisms [60]. This implies that there could be multiple acceptable models for describing globally observed dengue dynamics, none of which can easily be rejected and therefore should all be considered in assessing the mechanisms determining disease transmission [61–63]. Three major efforts that would help disentangle the dominant drivers of dengue are: 1) better estimates of model parameters, in particular the duration of cross-immunity and the strength of seasonal forcing; 2) improved understanding on the contribution of post-secondary infections to dengue transmission dynamics; and 3) additional, more detailed patterns, such as (i) time series of serotype-specific dengue cases and (ii) levels of sero-prevalence in populations. Some of these patterns may well differ across geographic regions.

Based on the sizes of the passing parameter distributions, a preference for the most complex 2-infection model was apparent (Table 4). Remarkably, the model that performs best across all models is the 4-infection model with CI only. This indicates that, in some instances, the use of multiple patterns for model selection can help filter out overly specialized models and fetch simple, more generalized models that perform better across different scales. Additionally, it helps reveal the impact of simplifying assumptions on model selection and parameterization, *i*.*e*. allowing for quaternary infections enables us to reveal a simpler model framework that outcompetes its 2-infection equivalent. Also, it sheds new light on the need for ADE in replicating dengue dynamics. The role of ADE is not supported when allowing quaternary and tertiary infections while it is preferred in the 2-infection case, with and without asymmetry in transmission rates.

The performance of the base-model is noteworthy, given that it does not include the explicit serotype interactions deemed necessary to replicate asynchronous serotype oscillations. However, there are two implicit serotype interactions that likely underlie this behaviour. First, in the 2-infection model, serotypes affect each other’s dynamics by causing complete immunity to all serotypes after recovery from the second infection. The observed collapse in model fit of the base-model when we allowed for tertiary and quaternary infections supports this hypothesis. However, the 4-infection base-model also generates desynchronized behaviour of serotypes albeit in a very sparse region of the parameter space. This may result from the other implicit serotype interaction as a result of constraining individuals from acquiring more than one infection at the same time. In other words, this second type of interaction arises because individuals infected with one serotype are cross-immune to the remaining serotypes for the duration of the infectious period. This interaction may be enough to underlie a few, sparse fits across the parameter space. Indeed, when the model is extended to include more than one concurrent infection, the out-of-sync oscillations observed in the 4-infection base-model disappear (S6 Fig).

An additional result revealed by the POM-approach is that model preference appears to be governed by the level of seasonal fluctuations. Namely, the support for models with CI is larger in low seasonal settings, whereas the inclusion of decomposed ADE is required to reproduce the observed dengue patterns in the presence of strong seasonal fluctuations (Fig 4B). However, when tertiary and quaternary infections are allowed, this pattern disappears and all models apart from the base-model reveal similar median values for seasonal forcing (S2bB Fig). Additionally, we observe that the estimates for the duration of cross-immune period differ markedly upon inclusion of ADE or when relaxing the two infection assumption. In fact, without the inclusion of ADE, the CI-only 2-infection model does not encapsulate the best estimate of the duration of the cross-immune period, as proposed by Reich et al. [21]. The CI-model in the 4-infection framework, does meet the values estimated. These findings highlight that improved understanding of the extent to which post-secondary dengue infections contribute to overall dengue transmission, may greatly aid in disentangling the dominant drivers of dengue dynamics.

The public health importance of knowing the processes governing dengue transmission in a specific setting is highlighted by our results on achieving transmission interruption by vector control. The results indicated that the vulnerability of the models to disruption in transmission at equal levels of R_0_, was driven by the immune interactions incorporated in the model, with CI increasing resistance in low transmission settings, while ADE has the opposite effect. It is common practice to favour the most parsimonious model when the candidate models are equally efficient; however, the differences in model resistance we found here suggest that it is prudent to be extra cautious while making such a decision. Given their decisive role in selecting and quantifying the predominant mechanisms as well as determining the projected effects of interventions, in addition to R_0_-estimates, obtaining improved, localised estimates of seasonal forcing and the duration of cross-immunity should be prioritized towards better-informing modelling endeavours.

While efforts to disentangle the extent to which internal and external drivers influence the dynamics of multi-serotype systems have been made [64], adequately incorporating both the complex serotype interactions as well as the effects of coupling and decoupling between seasonal forcing and incidence remains an important issue. This is more so because long time series for serotype-specific incidence and vector abundance are scarce and case data are distorted by misclassification and underreporting. The core of the POM approach lies in the appreciation that single data patterns (*e*.g. multi-annual signals) usually do not contain enough information to unambiguously identify the mechanism generating such patterns; additional patterns from data are needed to fit several model responses simultaneously [65]. As pointed out above, we have shown here that, even with sparse data and relatively wide criteria, POM can be a useful tool to distinguish between different conceptual models for capturing dengue dynamics and assessing their vulnerability to control.

While the use of multiple patterns enhances the process of model selection greatly, it is not always clear whether a model capable of replicating the observed patterns can react realistically to environmental perturbations. This may especially be pertinent here as the models are fitted to macroscopic data using the average behaviour of the dynamical system rather than lower level processes [24]. While the proposed framework could be extended to incorporate additional, lower level patterns, such as serotype driven variation in disease severity, age-distributions of sero-prevalence, or age at first infection, these are likely to vary across regions and would greatly enhance the parameter dimension to be studied, diminishing the transparency and insights gained into the distribution and behaviour of model parameters which is our main focus. Similarly, matching to multiple patterns may not be sufficient to overcome the suspicion that the models demonstrate unrealistic resistance to control, as over 40 weeks of interrupted transmission is required to bring about an 80% probability of success (Fig 6). The import factor prevents the models from showing unviable dynamic behaviour that results from unrealistically low levels of infections innate to ODE-systems in general and especially prevalent in models with ADE(x2), yet also enhances the resistance of models. While the absolute levels of control are thus of limited practical use, the overall conclusion of differential resistance is found to persist across models with a lower import factor as well (S8 Fig), highlighting a fundamental challenge arising from structural model uncertainty.

The criteria derived and used in this work may be subjective. By basing the criteria on current literature and the available data and keeping the characteristics broad, we aimed to limit such subjectivity. By focussing on patterns that are common across endemic regions, the derived patterns are inherently weaker than for a localised approach, yet the outcomes are more generalizable. The broadness of the characteristics does lead to decreased uniqueness (as model fits to dengue patterns can be found across the entire parameter space) [66] and a wide range of model behaviours (S4a–S4c Fig) [67]. To reduce subjectivity, we have used uniform distributions bounded by ranges informed by literature. For model calibration, too restricted ranges may underestimate the level of uncertainty around a parameter value, whereas in model selection, the proportion of passes is sensitive to the width of the range. Also, the comparison between the models with different numbers of sampled parameters has underlying difficulties. In more complex models, the passing parameter space may be underrepresented, giving rise to a local decrease in likelihood and wider parameter bounds [68]. However, given the small number of parameters and large number of parameter combinations examined, the severity of under-sampling in this exercise is limited. Finally, caution should be taken in judging the likelihood of models based on the number of passes, as no correction is made for the differential complexity between the models.

The six models examined were chosen based on their proven performance in the literature [13,15,19]. However, the models contain some inherent limitations. The limited persistence typical in highly seasonal models with (decomposed) ADE may in part result from the lack of stochasticity in the model [12,23]. Serotype persistence is also believed to be affected by the assumed symmetry in transmission rate and or virulence between serotypes [17]. We indeed observe less wild fluctuations upon the inclusion of asymmetry and a consequential increase in the fit of models with ADE (S4b Fig). Further, the inclusion of explicit vector dynamics has been found to increase the robustness of the system to changes in cross-immunity and ADE parameters, resulting in a larger parameter space with regular (1–2 year inter-epidemic periods) dynamics and moderate amplitude fluctuations [69]. Therefore, including vector population dynamics may affect the quantitative conclusions of this study, especially when high seasonal fluctuations are assumed. The inclusion of explicit vector dynamics would further allow for a more quantitative assessment of required control efforts, which will be a focus of future work.

Lastly, no long-term variation in parameter values was taken into account. Yet, fertility rates have decreased and life expectancy has gone up in most dengue endemic countries over the last decades [70]. Cummings et al. showed that a decrease in birth rate might result in a decrease in the force of infection and increase in the mean age of infection [71]. The same authors also demonstrate that this demographic shift may have induced prolonged multiannual oscillations [71]. Additionally, vector control has intensified over the years with varying success [72]. The on-and-off vector control is likely to act as a distorting factor in the estimation of the role of seasonality, as the climate driven signal in the incidence data may be weakened by these control measures. Therefore, ignoring on-going control measures may have had some influence in our model selection and predictions. Further research will focus on disentangling the complex interplay of dengue dynamics with non-stationary factors such as intervention efforts, demography and climate.

With the expanding spatial spread of dengue and the increase of frequency and size of outbreaks, understanding dengue disease dynamics and the consequences of control efforts (*e*.*g*. a near-future vaccine introduction) has become critically important. Indeed, the present work stresses that ignoring model uncertainty in prediction exercises can skew the impact of vector control substantially. It also emphasizes that the wider use of improved data-model assimilation approaches, such as the POM method, could play a significant role in overcoming this problem.

## Supporting Information

S1 Fig Outcome measures plane plots for the symmetric 2-infection (a), asymmetric 2-infection (b) and symmetric 4-infection model (c). Analysis of the parameter space of each model structure (with ADE = antibody dependent enhancement, CI = cross-immunity) for seasonality (β_1_) and the basic reproduction number (R_0_). From top to bottom, outcomes are measured with respect to (A) mean inter-peak period, (B) presence of multi-annual signal (red = present, blue = absent), (C) duration of serotype replacement, (D) single serotype emergence and (E) absence of phase-locking (red = absent, blue = present).
(PDF)

S2 Fig Model parameter distributions for the asymmetric 2-infection (a) and symmetric 4-infection model (b). Parameter distributions for passing parameter sets (G) for different model hypotheses (with ADE = antibody dependent enhancement, CI = cross-immunity). The vertical lines depict the median values for each distribution with the colours indicating the corresponding model hypothesis.
(PDF)

S3 Fig Correlation matrix full model for the symmetric 2-infection (a), asymmetric 2-infection (b) and symmetric 4-infection model (c). Correlation between passing parameters in full model (ADEx2+CI) with red numbers depicting a significant correlation coefficient. The respective parameter distributions are shown on the diagonal.
(PDF)

S4 Fig Qualitative comparison observed dengue case data and passing model simulations for the symmetric 2-infection (a), asymmetric 2-infection (b) and symmetric 4-infection model (c). Qualitative comparison between observed dengue incidence data and model simulations at median levels of seasonal forcing. Dengue incidence data from Trinidad and Tobago (1997–2009) were duplicated for comparison with model simulations (A). The dotted vertical lines indicate the length of the original dataset. Other parameter values are derived at random from the passing parameter distribution G with: (**a**) the symmetric 2-infection model: (A)β_0_ = 344, β_1_ = 0.1, *α*_*SUS*_ = 1, *α*_*TRANS*_ = 1, *ρ* = NA (B), β_0_ = 204, β_1_ = 0.06, *α*_*SUS*_ = 1, *α*_*TRANS*_ = 1, *ρ* = 2.8 (C), β_0_ = 240, β_1_ = 0.11, *α*_*SUS*_ = 1.28, *α*_*TRANS*_ = 1, *ρ* = NA (D), β_0_ = 276, β_1_ = 0.05, *α*_*SUS*_ = 1.64, *α*_*TRANS*_ = 1, *ρ* = 2.0 (E), β_0_ = 228, β_1_ = 0.16, *α*_*SUS*_ = 1.05, *α*_*TRANS*_ = 2.23, *ρ* = NA (F) and β_0_ = 220, β_1_ = 0.12, *α*_*SUS*_ = 1.61, *α*_*TRANS*_ = 1.39, *ρ* = 2.37 (G) (**b**) asymmetric 2-infection model: (A)β_0_ = 252, β_1_ = 0.11, *α*_*SUS*_ = 1, *α*_*TRANS*_ = 1, *ρ* = NA (B), β_0_ = 384, β_1_ = 0.24, *α*_*SUS*_ = 1, *α*_*TRANS*_ = 1, *ρ* = 1.5 (C), β_0_ = 323, β_1_ = 0.26, *α*_*SUS*_ = 2.23, *α*_*TRANS*_ = 1, *ρ* = NA (D), β_0_ = 279, β_1_ = 0.3, *α*_*SUS*_ = 1.86, *α*_*TRANS*_ = 1.26, *ρ* = 2.0 (E), β_0_ = 228, β_1_ = 0.16, *α*_*SUS*_ = 1.05, *α*_*TRANS*_ = 2.23, *ρ* = NA (F) and β_0_ = 327, β_1_ = 0.30, *α*_*SUS*_ = 1.16, *α*_*TRANS*_ = 1.54, *ρ* = 2.35 (G) (**c**) symmetric 4-infection model: (A)β_0_ = 249, β_1_ = 0.07, *α*_*SUS*_ = 1, *α*_*TRANS*_ = 1, *ρ* = NA (B), β_0_ = 308, β_1_ = 0.29, *α*_*SUS*_ = 1, *α*_*TRANS*_ = 1, *ρ* = 1.26 (C), β_0_ = 161, β_1_ = 0.09, *α*_*SUS*_ = 2.08, *α*_*TRANS*_ = 1, *ρ* = NA (D), β_0_ = 188, β_1_ = 0.13, *α*_*SUS*_ = 2.17, *α*_*TRANS*_ = 1, *ρ* = 1.0 (E), β_0_ = 198, β_1_ = 0.17, *α*_*SUS*_ = 1.12, *α*_*TRANS*_ = 1.40, *ρ* = NA (F) and β_0_ = 125, β_1_ = 0.29, *α*_*SUS*_ = 1.90, *α*_*TRANS*_ = 1.68, *ρ* = 1.04 (G) (with β_0_ = mean transmission rate, β_1_ = seasonal forcing, *α*_*SUS*_ = susceptibility enhancement, *α*_*TRANS*_ = transmissibility enhancement, *ρ* = 1/duration of cross-immunity)
(PDF)

S5 Fig Principal component analysis for the asymmetric 2-infection (a) and symmetric 4-infection model (b). Principal component analysis of passing parameter space (G) of the full model (ADEx2+CI). The pie charts show the contribution of the parameters to each component.
(PDF)

S6 Fig Comparative model simulations of 4-infection base-model with and without concurrent infections. Model simulations at passing parameter sets of the 4-infection base-model without concurrent infections (top row) and with concurrent infection (bottom row). The colours indicate different serotypes. Parameter values are: (left)β_0_ = 249, β_1_ = 0.07, *α*_*SUS*_ = 1, *α*_*TRANS*_ = 1, *ρ* = NA (middle), β_0_ = 333, β_1_ = 0.07, *α*_*SUS*_ = 1, *α*_*TRANS*_ = 1, *ρ* = NA (right), β_0_ = 263, β_1_ = 0.14, *α*_*SUS*_ = 1, *α*_*TRANS*_ = 1, *ρ* = NA
(TIF)

S7 Fig Vulnerability to control as a function of model parameters. Required duration (weeks/year) for achieving successful control is shown with respect to fitted model parameters. Different model hypotheses are (from top to bottom): base (A), CI (B), ADE (C), ADE+CI (D), ADEx2 (E), and ADEx2+CI (F), with ADE = antibody dependent enhancement, CI = cross-immunity. Model parameters assessed are (from left to right): (A) the transmission rate (β_0_), (B) seasonality (β_1_), (C) enhanced susceptibility (α_SUS_), (D) enhanced infectiousness (α_TRANS_), and (E) cross-immunity (ρ).
(TIF)

S8 Fig Effect of import factor on vulnerability to control. Probability of successful control (a maximum of 1 outbreak during 30 years) given different durations (10, 20, and 30 weeks/year) of consecutive control (temporary reduction of transmission: *β*_0_(1−90%)for different model hypotheses (with ADE = antibody dependent enhancement, CI = cross-immunity). The probability is defined as the proportion of the passing parameter sets (*G*_*i*_) that reach successful control. Here *i* refers to the six models, shown by the individual keys. The top row (A, B, and C) shows the results for the default import rate of 1e-10. The bottom row (D, E, and F) shows results for a decreased import rate of 1e-12. The probability of successful control for the Base-model and the CI-model in the default scenario are zero, as can also be seen in Fig 6.
(TIF)

S1 Table Sensitivity analysis of model fits on all model parameterizations including interactions. Logistic regression model coefficients with pattern match as binary response variables and the parameters (scaled 0–1) as independent variables. Red are high coefficient values (>|3|).
(XLSX)

S1 Text System of differential equations for 4-infection model. (DOCX)

S2 Text Proof R_0_. (DOCX)
