# Supplementary material for: The Role of Serotype Interactions and Seasonality in Dengue Model Selection and Control: Insights from a Pattern Matching Approach
Source: PLoS Negl Trop Dis. 2016 May 9;10(5):e0004680. doi: 10.1371/journal.pntd.0004680 (PMC4861330; doi:10.1371/journal.pntd.0004680)
Supplement: S1 Fig — Analysis of the parameter space of each model structure (with ADE = antibody dependent enhancement, CI = cross-immunity) for seasonality (β1) and the basic reproduction number (R0). From top to bottom, outcomes are measured with respect to (A) mean inter-peak period, (B) presence of multi-annual signal (red = present, blue = absent), (C) duration of serotype replacement, (D) single serotype emergence and (E) absence of phase-locking (red = absent, blue = present). (PDF) [file pntd.0004680.s001.pdf]

(a) The symmetric 2-infection model:

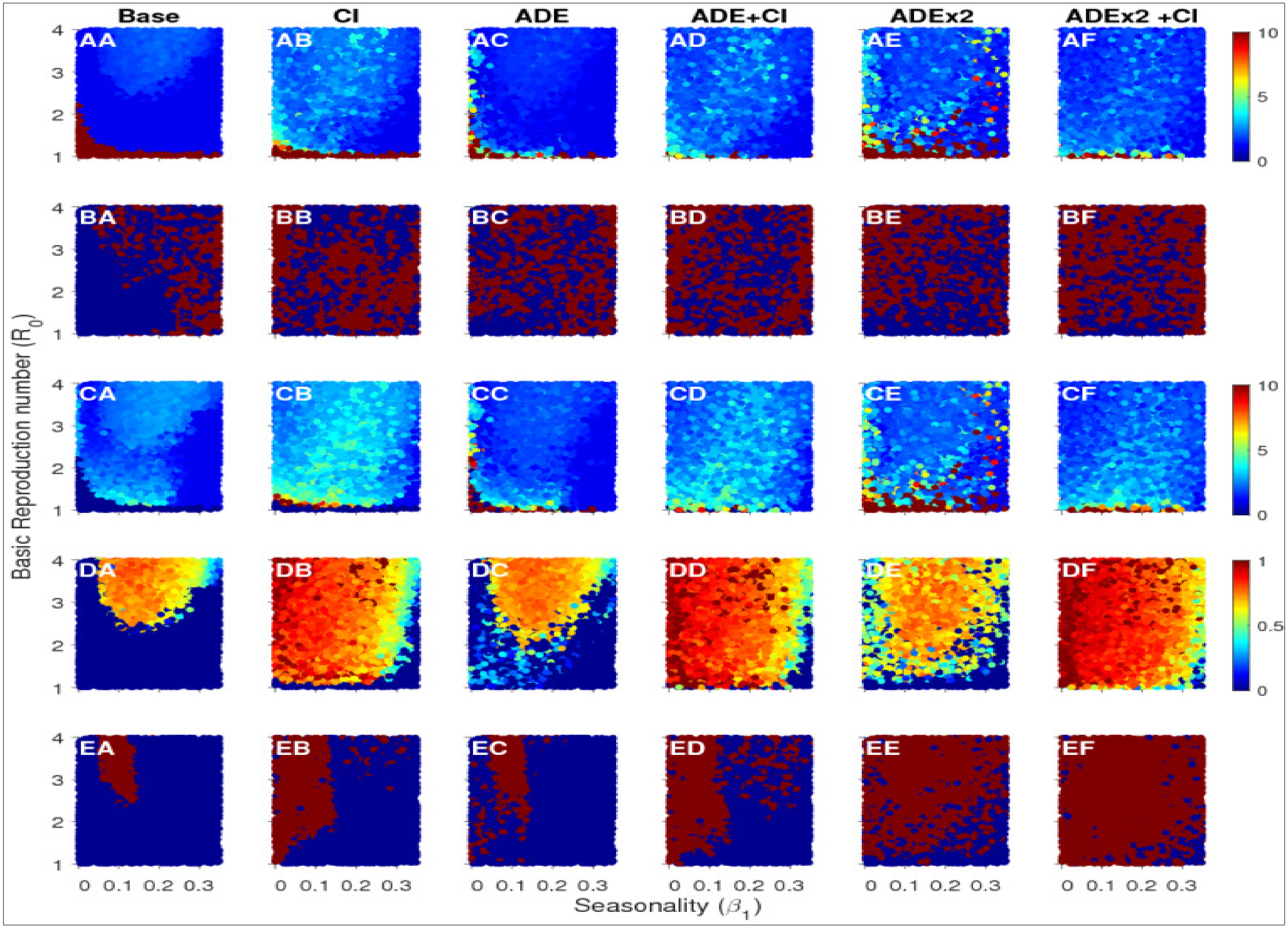

(b) The asymmetric 2-infection model:

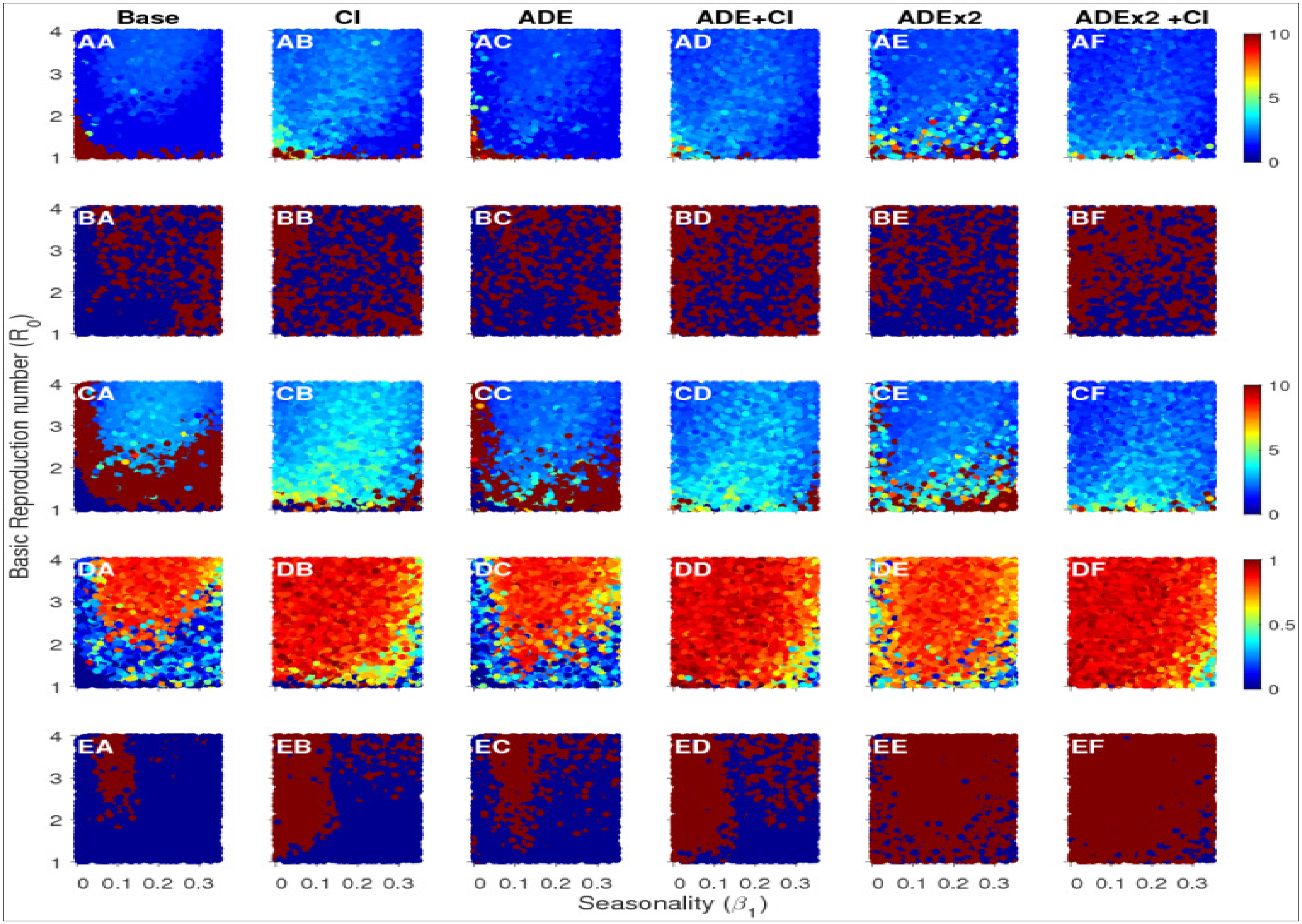

(c) The symmetric 4-infection model

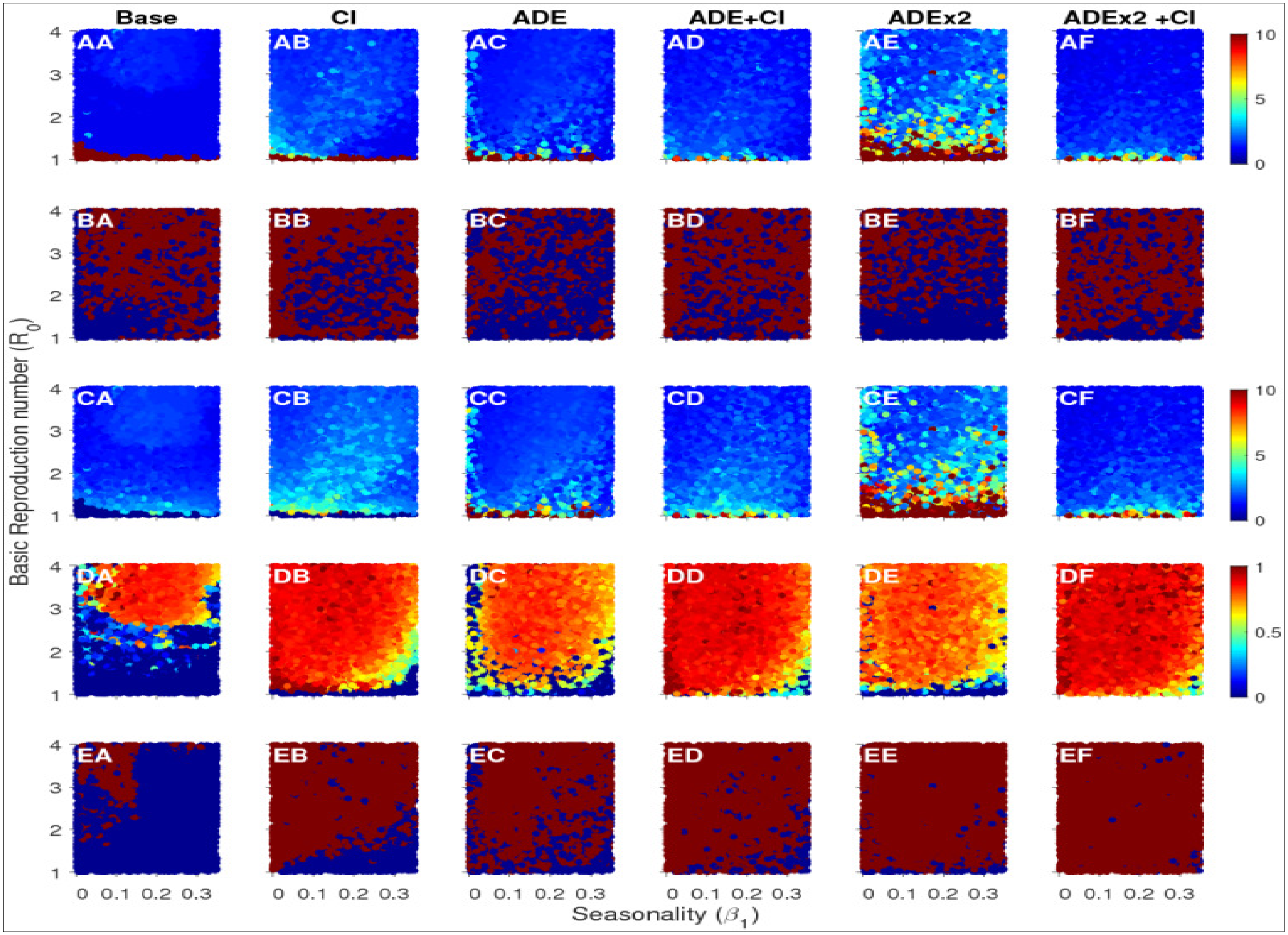

S1 Fig: Outcome measures plane plots for the symmetric 2-infection (a), asymmetric 2-infection (b) and symmetric 4-infection model (c). Analysis of the parameter space of each model structure (with ADE=antibody dependent enhancement, CI=cross-immunity) for seasonality (β_1) and the basic reproduction number (R_0). From top to bottom, outcomes are measured with respect to (A) mean inter-peak period, (B) presence of multi-annual signal (red = present, blue = absent), (C) duration of serotype replacement, (D) single serotype emergence and (E) absence of phase locking (red = absent, blue = present).
