# Supplementary material for: The Role of Serotype Interactions and Seasonality in Dengue Model Selection and Control: Insights from a Pattern Matching Approach
Source: PLoS Negl Trop Dis. 2016 May 9;10(5):e0004680. doi: 10.1371/journal.pntd.0004680 (PMC4861330; doi:10.1371/journal.pntd.0004680)
Supplement: S2 Fig — Parameter distributions for passing parameter sets (G) for different model hypotheses (with ADE = antibody dependent enhancement, CI = cross-immunity). The vertical lines depict the median values for each distribution with the colours indicating the corresponding model hypothesis. (PDF) [file pntd.0004680.s002.pdf]

(a) The asymmetric 2-infection model:

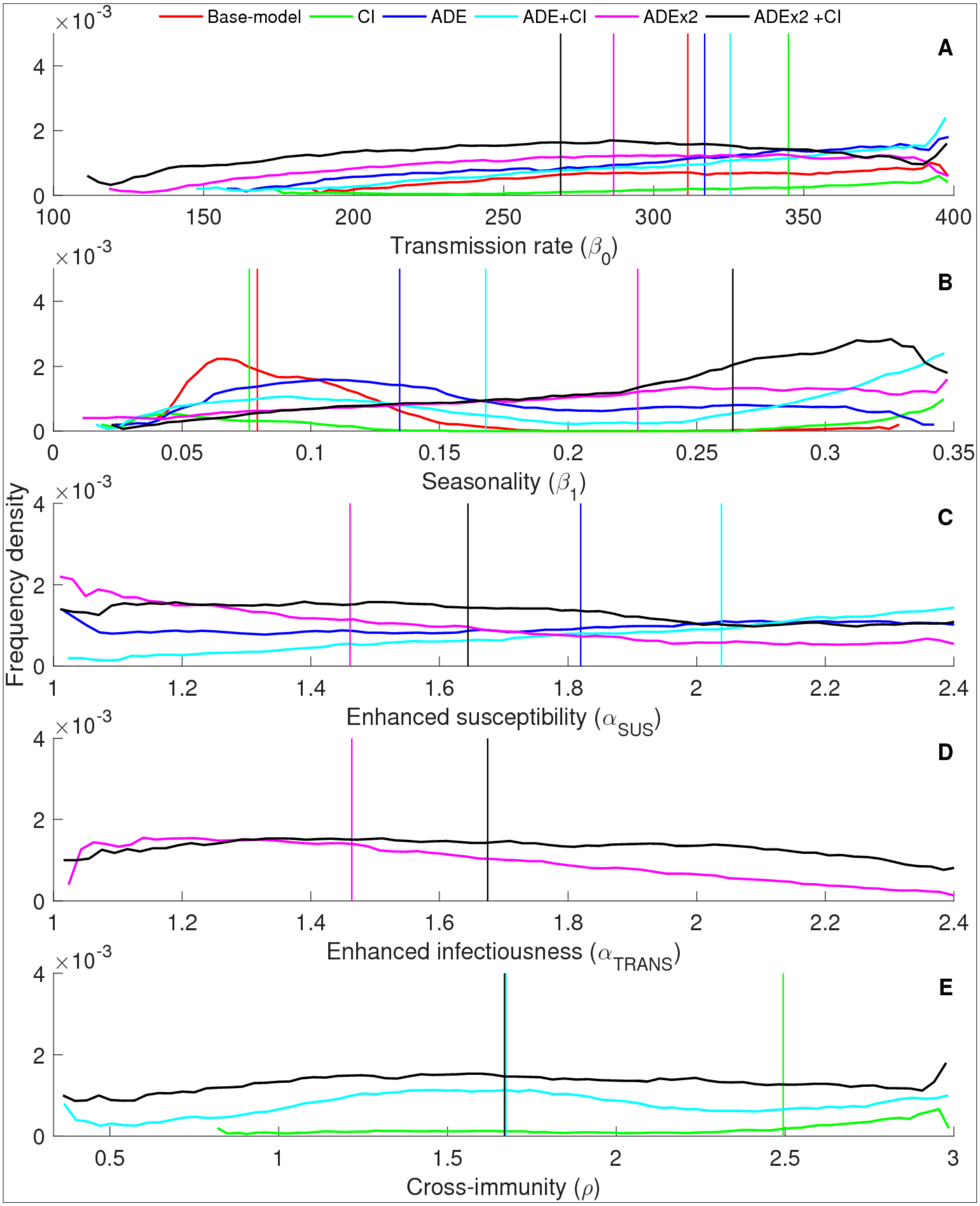

(b) The symmetric 4-infection model:

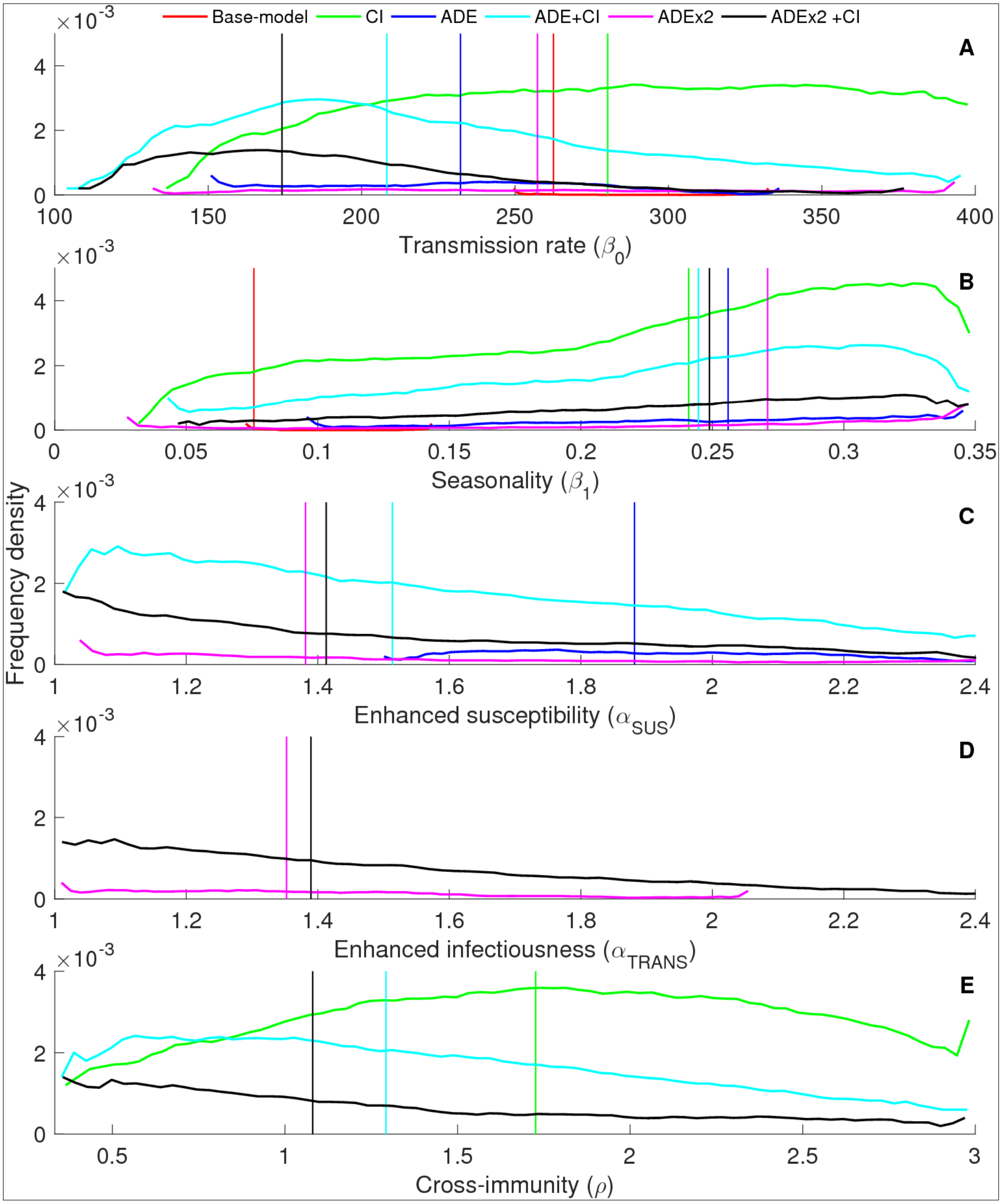

S2 Fig: Model parameter distributions for the asymmetric 2-infection (a) and symmetric 4-infection model (b). Parameter distributions for passing parameter sets (G) for different model hypotheses (with ADE=antibody dependent enhancement, CI=cross-immunity). The vertical lines depict the median values for each distribution with the colours indicating the corresponding model hypothesis**.**
