# Supplementary material for: The Role of Serotype Interactions and Seasonality in Dengue Model Selection and Control: Insights from a Pattern Matching Approach
Source: PLoS Negl Trop Dis. 2016 May 9;10(5):e0004680. doi: 10.1371/journal.pntd.0004680 (PMC4861330; doi:10.1371/journal.pntd.0004680)
Supplement: S3 Fig — Correlation between passing parameters in full model (ADEx2+CI) with red numbers depicting a significant correlation coefficient. The respective parameter distributions are shown on the diagonal. (PDF) [file pntd.0004680.s003.pdf]

**(a) The symmetric 2-infection model:**

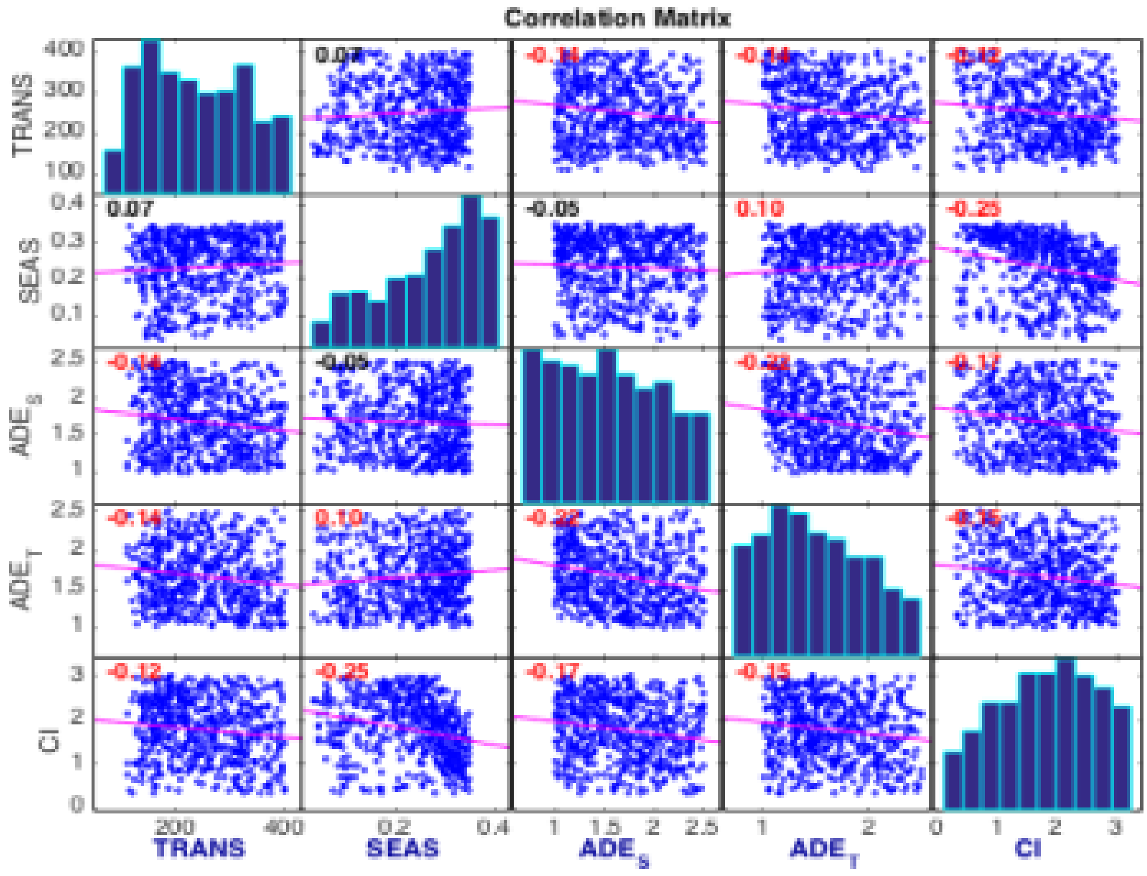

**(b) The asymmetric 2-infection model:**

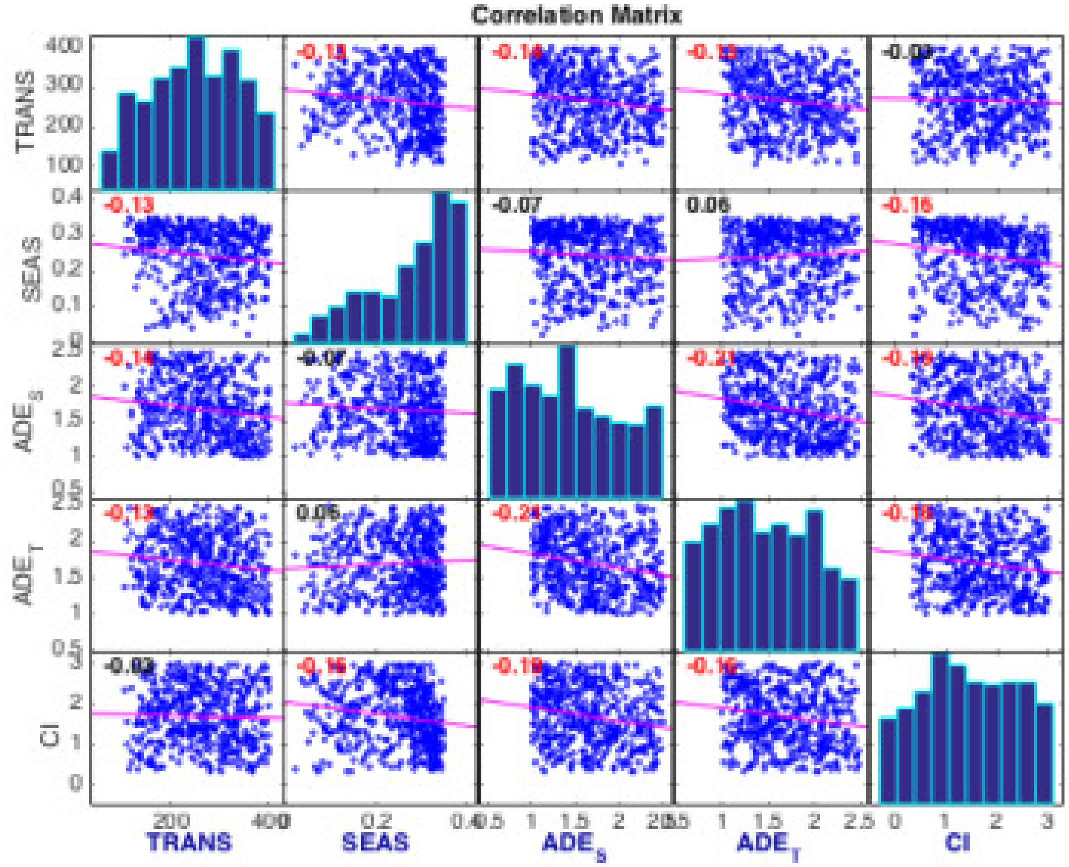

**(c) The symmetric 4-infection model:**

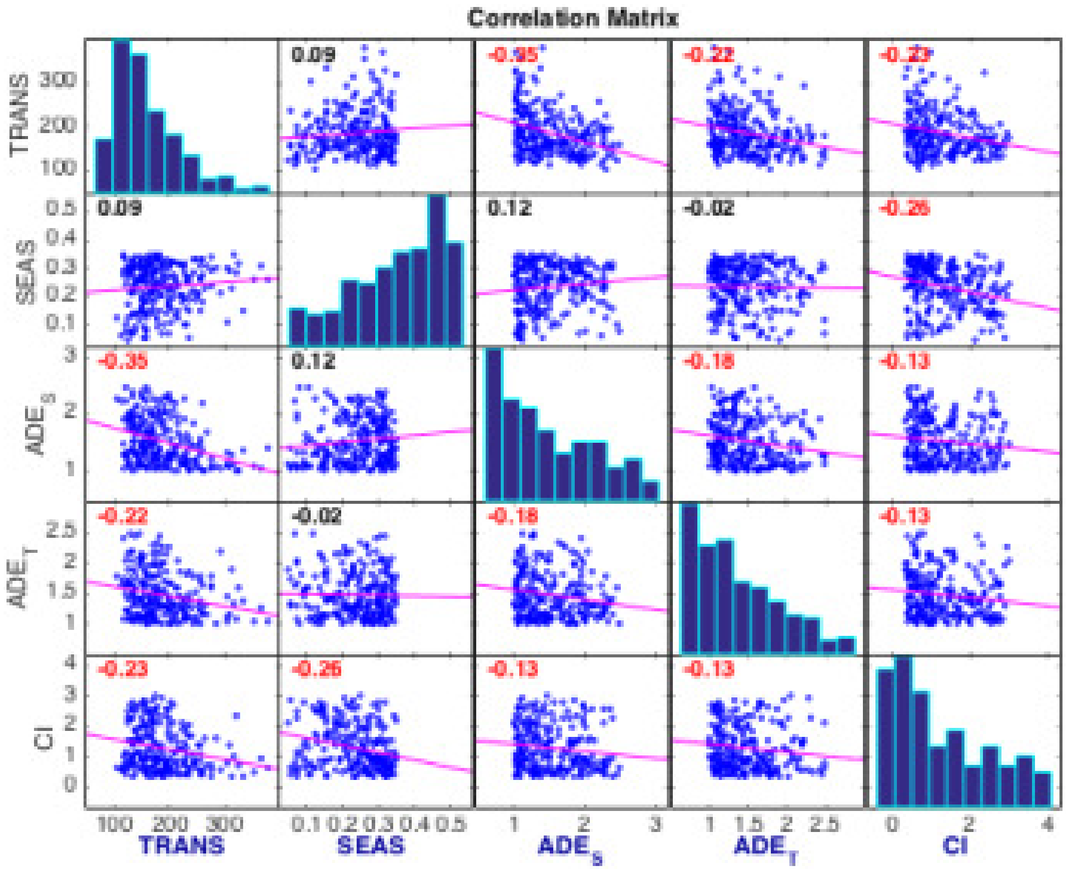


**S3 Fig: Correlation matrix full model for the symmetric 2-infection (a), asymmetric 2-infection (b) and symmetric 4-infection model (c).** Correlation between passing parameters in full model (ADEx2+CI) with red numbers depicting a significant correlation coefficient. The respective parameter distributions are shown on the diagonal.
