# Supplementary material for: The Role of Serotype Interactions and Seasonality in Dengue Model Selection and Control: Insights from a Pattern Matching Approach
Source: PLoS Negl Trop Dis. 2016 May 9;10(5):e0004680. doi: 10.1371/journal.pntd.0004680 (PMC4861330; doi:10.1371/journal.pntd.0004680)
Supplement: S4 Fig — Qualitative comparison between observed dengue incidence data and model simulations at median levels of seasonal forcing. Dengue incidence data from Trinidad and Tobago (1997–2009) were duplicated for comparison with model simulations (A). The dotted vertical lines indicate the length of the original dataset. Other parameter values are derived at random from the passing parameter distribution G with: (a) the symmetric 2-infection model: (A)β0 = 344, β1 = 0.1, αSUS = 1, αTRANS = 1, ρ = NA (B), β0 = 204, β1 = 0.06, αSUS = 1, αTRANS = 1, ρ = 2.8 (C), β0 = 240, β1 = 0.11, αSUS = 1.28, αTRANS = 1, ρ = NA (D), β0 = 276, β1 = 0.05, αSUS = 1.64, αTRANS = 1, ρ = 2.0 (E), β0 = 228, β1 = 0.16, αSUS = 1.05, αTRANS = 2.23, ρ = NA (F) and β0 = 220, β1 = 0.12, αSUS = 1.61, αTRANS = 1.39, ρ = 2.37 (G) (b) asymmetric 2-infection model: (A)β0 = 252, β1 = 0.11, αSUS = 1, αTRANS = 1, ρ = NA (B), β0 = 384, β1 = 0.24, αSUS = 1, αTRANS = 1, ρ = 1.5 (C), β0 = 323, β1 = 0.26, αSUS = 2.23, αTRANS = 1, ρ = NA (D), β0 = 279, β1 = 0.3, αSUS = 1.86, αTRANS = 1.26, ρ = 2.0 (E), β0 = 228, β1 = 0.16, αSUS = 1.05, αTRANS = 2.23, ρ = NA (F) and β0 = 327, β1 = 0.30, αSUS = 1.16, αTRANS = 1.54, ρ = 2.35 (G) (c) symmetric 4-infection model: (A)β0 = 249, β1 = 0.07, αSUS = 1, αTRANS = 1, ρ = NA (B), β0 = 308, β1 = 0.29, αSUS = 1, αTRANS = 1, ρ = 1.26 (C), β0 = 161, β1 = 0.09, αSUS = 2.08, αTRANS = 1, ρ = NA (D), β0 = 188, β1 = 0.13, αSUS = 2.17, αTRANS = 1, ρ = 1.0 (E), β0 = 198, β1 = 0.17, αSUS = 1.12, αTRANS = 1.40, ρ = NA (F) and β0 = 125, β1 = 0.29, αSUS = 1.90, αTRANS = 1.68, ρ = 1.04 (G) (with β0 = mean transmission rate, β1 = seasonal forcing, αSUS = susceptibility enhancement, αTRANS = transmissibility enhancement, ρ = 1/duration of cross-immunity) (PDF) [file pntd.0004680.s004.pdf]

(a) **The symmetric 2-infection model:**

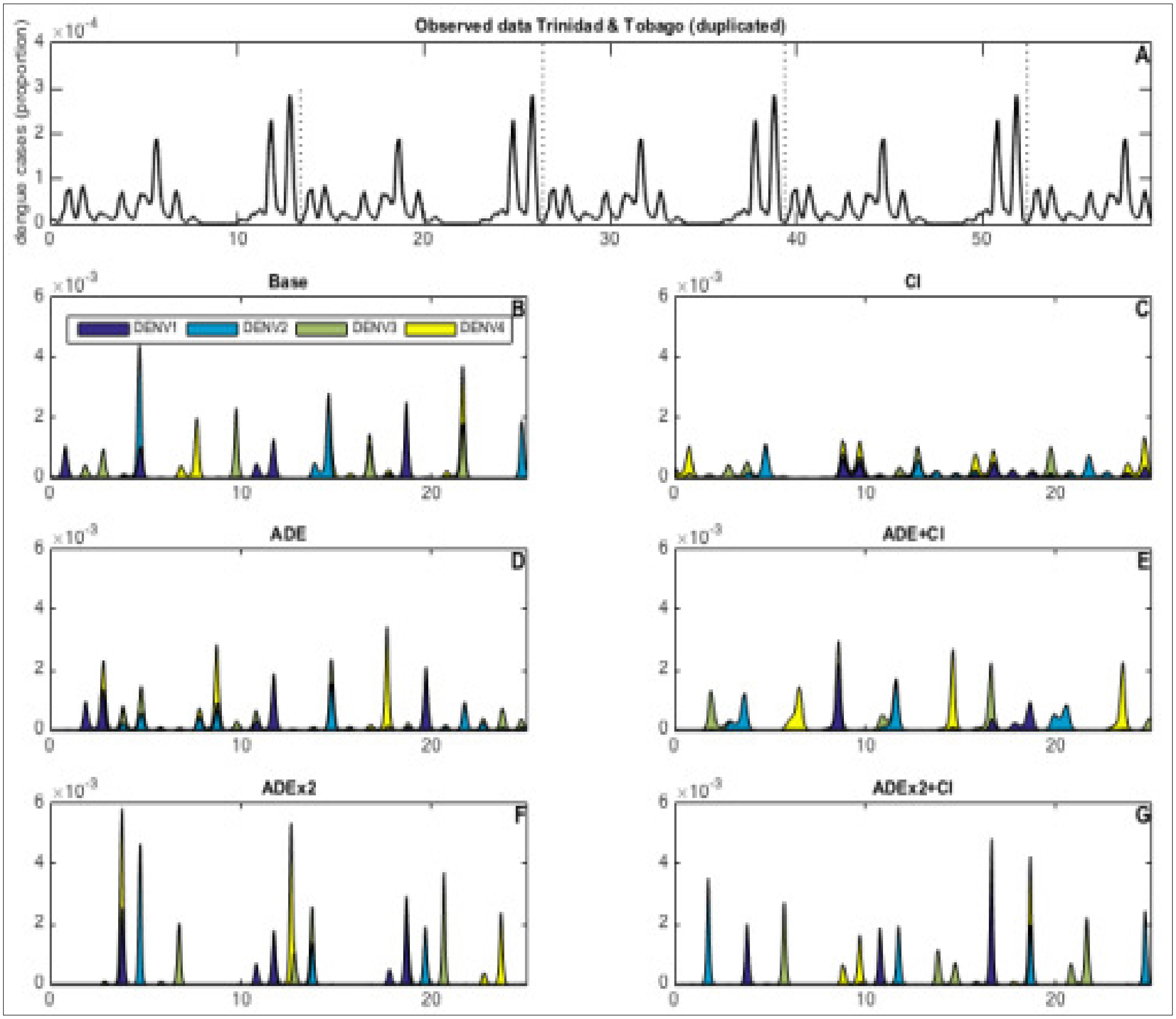

(b) **The asymmetric 2-infection model:**

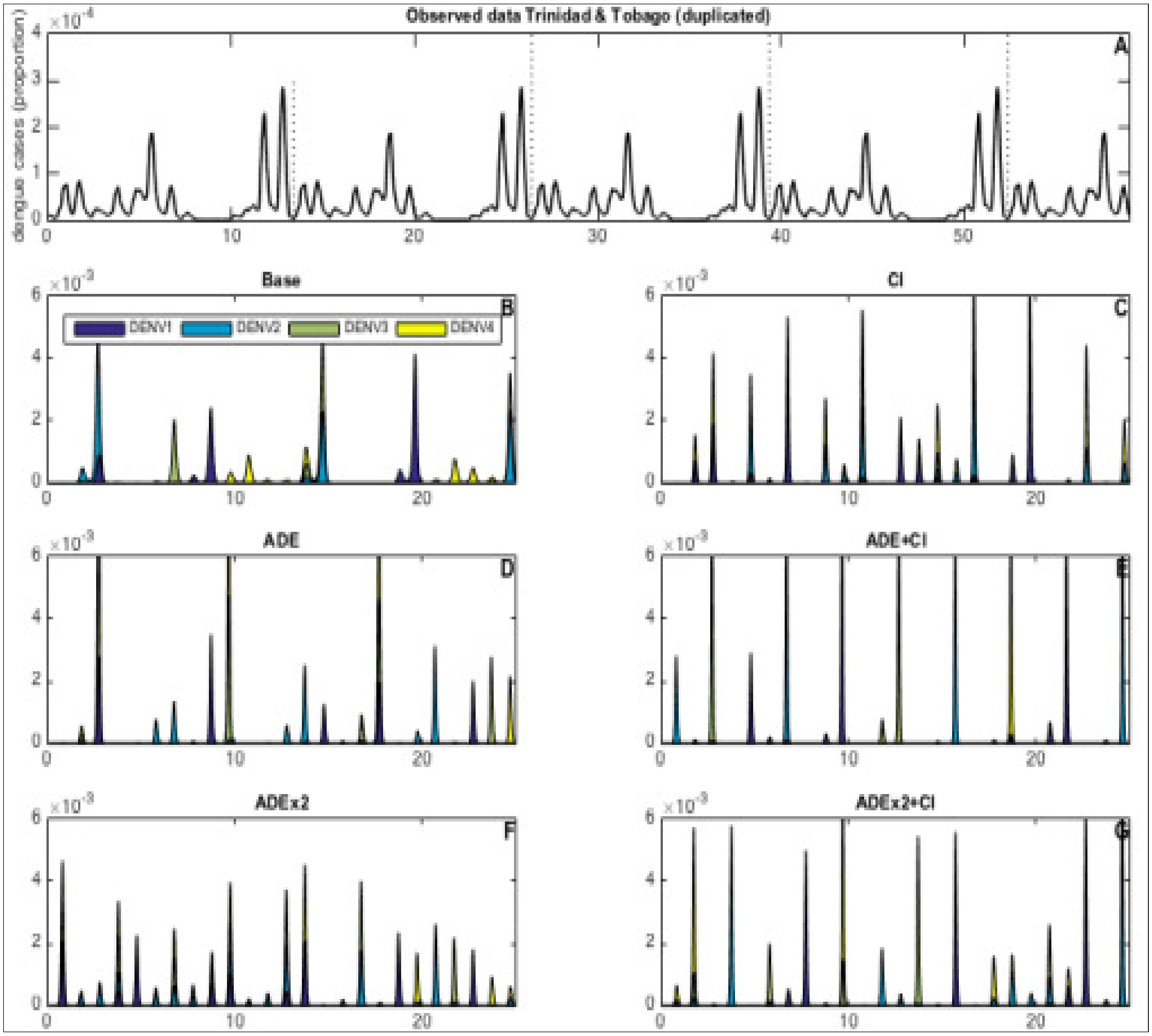

(c) The symmetric 4-infection model:

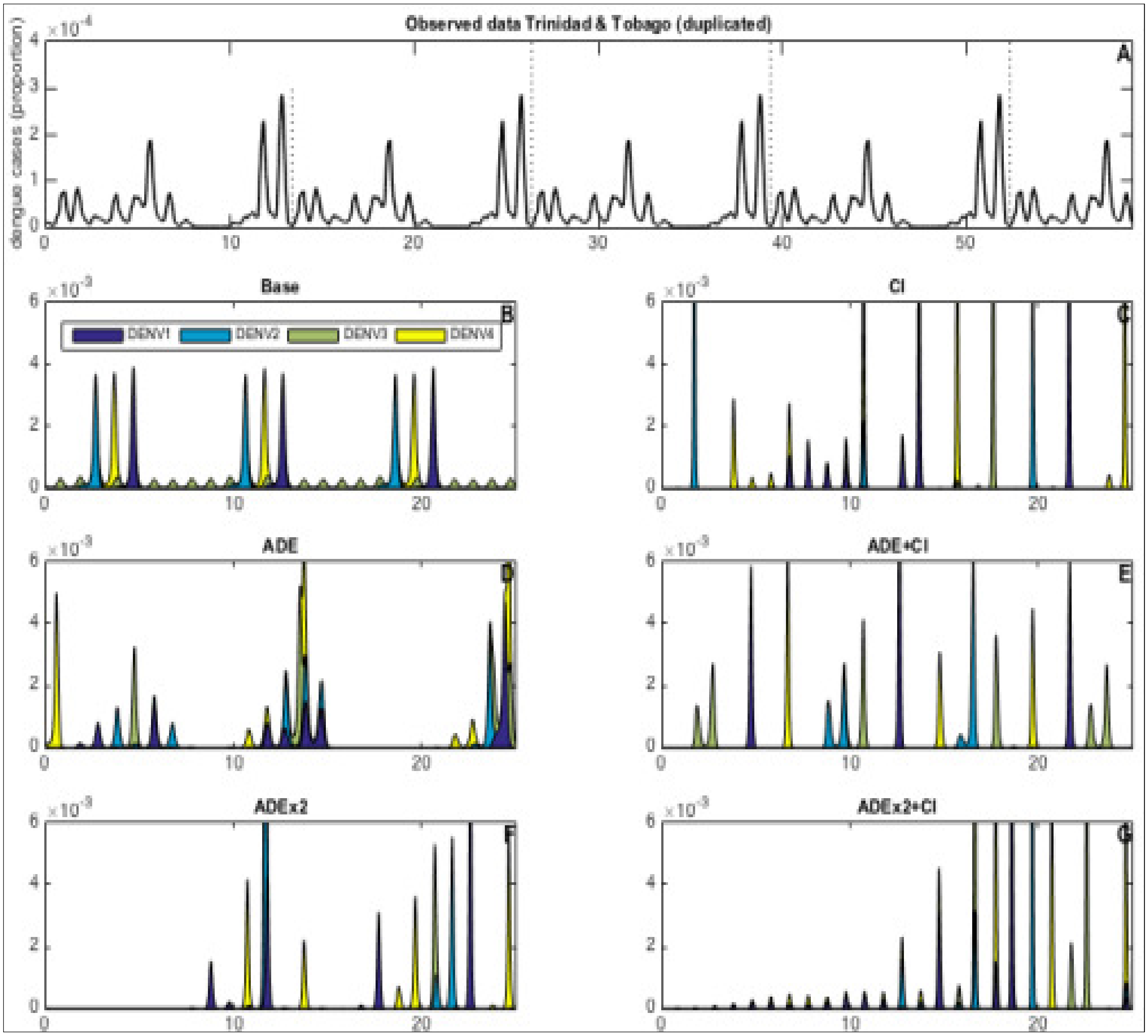

S4 Fig: Qualitative comparison observed dengue case data and passing model simulations for the symmetric 2-infection (a), asymmetric 2-infection (b) and symmetric 4-infection model (c). Qualitative comparison between observed dengue incidence data and model simulations at median levels of seasonal forcing. Dengue incidence data from Trinidad and Tobago (1997-2009) were duplicated for comparison with model simulations (A). The dotted vertical lines indicate the length of the original dataset. Other parameter values are derived at random from the passing parameter distribution G with: (**a**) 2-infection symmetric model: (A)β_0=344, β_1 =0.1, α_{SUS} =1, α_{TRANS} =1, ρ=NA (B), β_0=204, β_1 =0.06, α_{SUS} =1, α_{TRANS} =1, ρ=2.8 (C), β_0=240, β_1 =0.11, α_{SUS} =1.28, α_{TRANS} =1, ρ=NA (D), β_0=276, β_1 =0.05, α_{SUS} =1.64, α_{TRANS} =1, ρ=2.0 (E), β_0=228, β_1 = 0.16, α_{SUS} =1.05, α_{TRANS} =2.23, ρ=NA (F)

and β_0=220, β_1 =0.12, α_{SUS} =1.61, α_{TRANS} =1.39, ρ=2.37 (G); (**b**) asymmetric 2-infection model: (A)β_0=252, β_1 =0.11, α_{SUS} =1, α_{TRANS} =1, ρ=NA (B), β_0=384, β_1 =0.24, α_{SUS} =1, α_{TRANS} =1, ρ=1.5 (C), β_0=323, β_1 =0.26, α_{SUS} =2.23, α_{TRANS} =1, ρ=NA (D), β_0=279, β_1 =0.3, α_{SUS} =1.86, α_{TRANS} =1.26, ρ=2.0 (E), β_0=228, β_1 = 0.16, α_{SUS} =1.05, α_{TRANS} =2.23, ρ=NA (F) and β_0=327, β_1 =0.30, α_{SUS} =1.16, α_{TRANS} =1.54, ρ=2.35 (G); (**c**) 4-infection model: (A)β_0=249, β_1 =0.07, α_{SUS} =1, α_{TRANS} =1, ρ=NA (B), β_0=308, β_1 =0.29, α_{SUS} =1, α_{TRANS} =1, ρ=1.26 (C), β_0=161, β_1 =0.09, α_{SUS} =2.08, α_{TRANS} =1, ρ=NA (D), β_0=188, β_1 =0.13, α_{SUS} =2.17, α_{TRANS} =1, ρ=1.0 (E), β_0=198, β_1 = 0.17, α_{SUS} =1.12, α_{TRANS} =1.40, ρ=NA (F) and β_0=125, β_1 =0.29, α_{SUS} =1.90, α_{TRANS} =1.68, ρ=1.04 (G); with β_0 = mean transmission rate, β_1 = seasonal forcing, α_{SUS} = susceptibility enhancement, α_{TRANS} = transmissibility enhancement, ρ = 1/duration of cross-immunity.
