# Supplementary material for: The Role of Serotype Interactions and Seasonality in Dengue Model Selection and Control: Insights from a Pattern Matching Approach
Source: PLoS Negl Trop Dis. 2016 May 9;10(5):e0004680. doi: 10.1371/journal.pntd.0004680 (PMC4861330; doi:10.1371/journal.pntd.0004680)
Supplement: S5 Fig — Principal component analysis of passing parameter space (G) of the full model (ADEx2+CI). The pie charts show the contribution of the parameters to each component. (PDF) [file pntd.0004680.s005.pdf]

(a) The asymmetric 2-infection model:

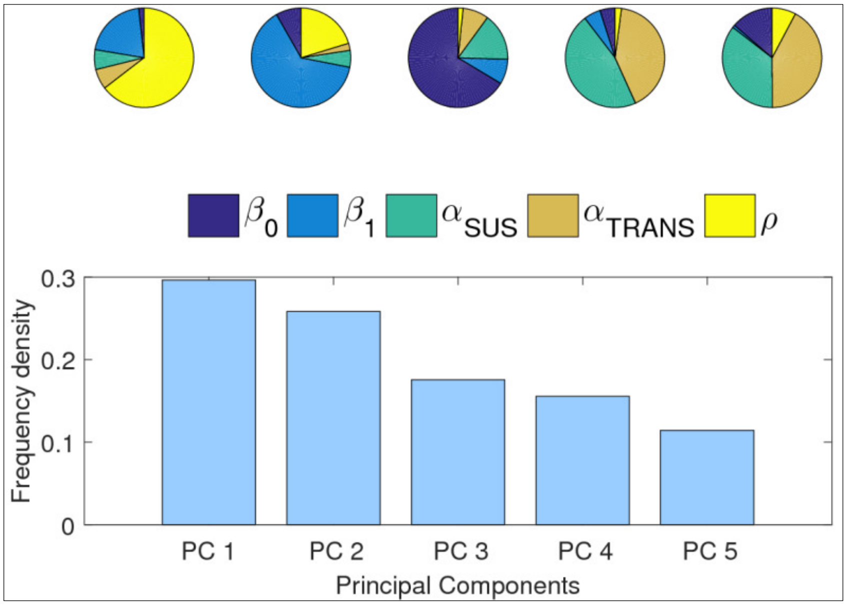

(b) The symmetric 4-infection model:

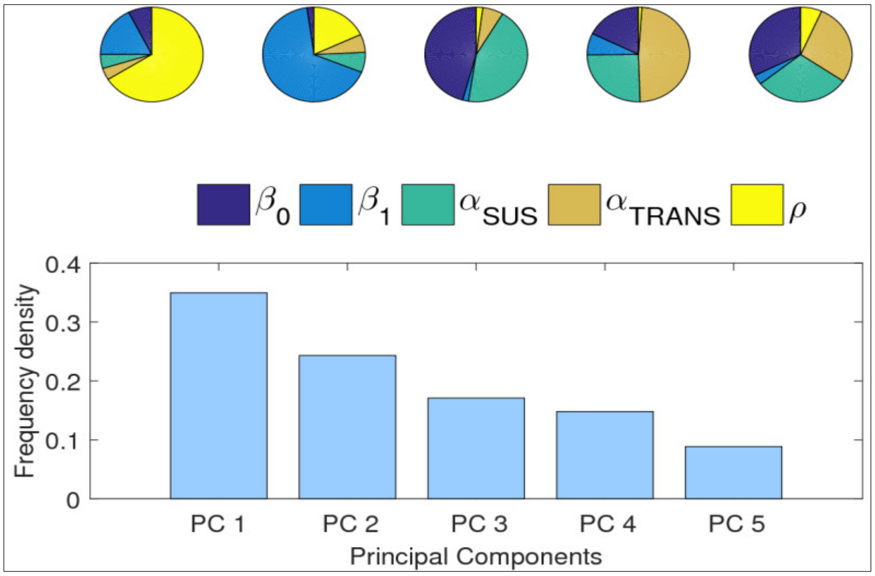

S5 Fig: Principal component analysis for the asymmetric 2-infection (a) and symmetric 4-infection model (b). Principal component analysis of passing parameter space (G) of the full model (ADEx2+CI). The pie charts show the contribution of the parameters to each component.
