# Supplementary figures and images for: The Role of Serotype Interactions and Seasonality in Dengue Model Selection and Control: Insights from a Pattern Matching Approach
Source: PLoS Negl Trop Dis. 2016 May 9;10(5):e0004680. doi: 10.1371/journal.pntd.0004680 (PMC4861330; doi:10.1371/journal.pntd.0004680)

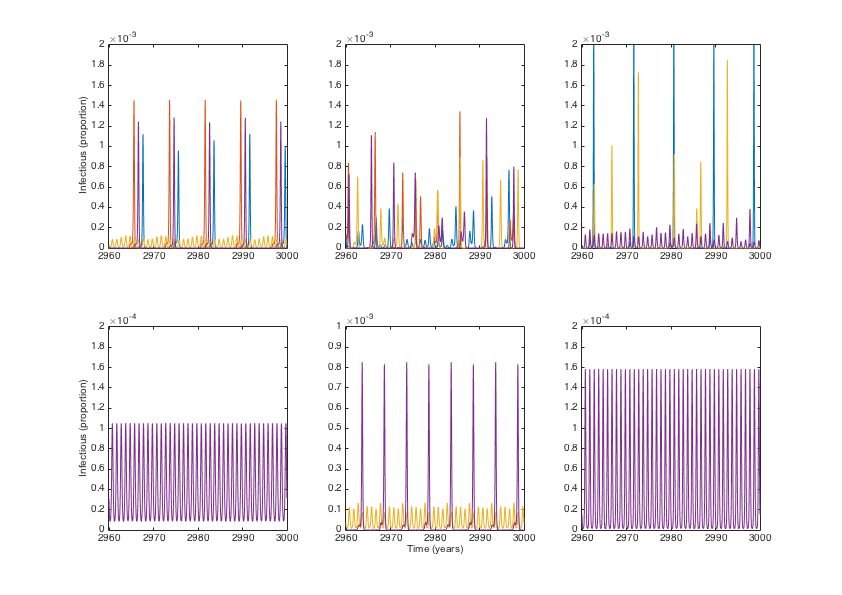

Supplement: S6 Fig — Model simulations at passing parameter sets of the 4-infection base-model without concurrent infections (top row) and with concurrent infection (bottom row). The colours indicate different serotypes. Parameter values are: (left)β0 = 249, β1 = 0.07, αSUS = 1, αTRANS = 1, ρ = NA (middle), β0 = 333, β1 = 0.07, αSUS = 1, αTRANS = 1, ρ = NA (right), β0 = 263, β1 = 0.14, αSUS = 1, αTRANS = 1, ρ = NA (TIF) [file pntd.0004680.s006.tif]

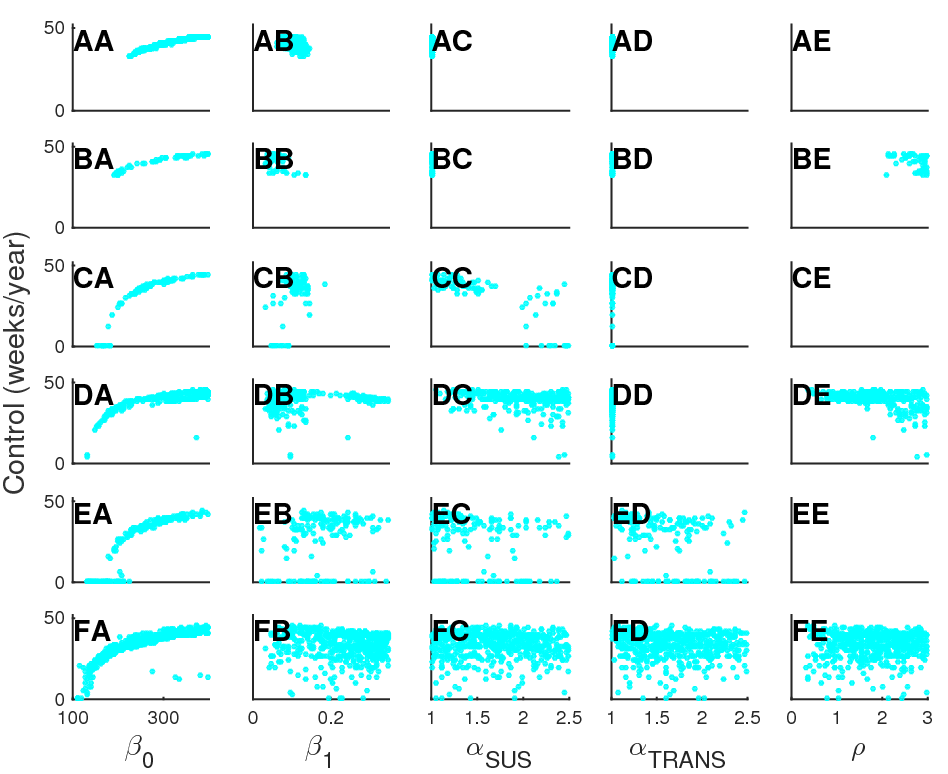

Supplement: S7 Fig — Required duration (weeks/year) for achieving successful control is shown with respect to fitted model parameters. Different model hypotheses are (from top to bottom): base (A), CI (B), ADE (C), ADE+CI (D), ADEx2 (E), and ADEx2+CI (F), with ADE = antibody dependent enhancement, CI = cross-immunity. Model parameters assessed are (from left to right): (A) the transmission rate (β0), (B) seasonality (β1), (C) enhanced susceptibility (αSUS), (D) enhanced infectiousness (αTRANS), and (E) cross-immunity (ρ). (TIF) [file pntd.0004680.s007.tif]

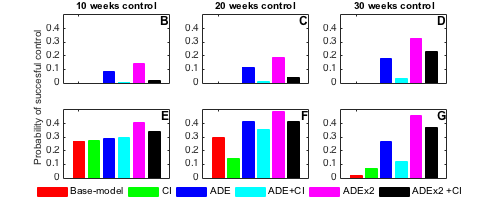

Supplement: S8 Fig — Probability of successful control (a maximum of 1 outbreak during 30 years) given different durations (10, 20, and 30 weeks/year) of consecutive control (temporary reduction of transmission: β0(1−90%)for different model hypotheses (with ADE = antibody dependent enhancement, CI = cross-immunity). The probability is defined as the proportion of the passing parameter sets (Gi) that reach successful control. Here i refers to the six models, shown by the individual keys. The top row (A, B, and C) shows the results for the default import rate of 1e-10. The bottom row (D, E, and F) shows results for a decreased import rate of 1e-12. The probability of successful control for the Base-model and the CI-model in the default scenario are zero, as can also be seen in Fig 6. (TIF) [file pntd.0004680.s008.tif]
