# Supplementary material for: The Role of Serotype Interactions and Seasonality in Dengue Model Selection and Control: Insights from a Pattern Matching Approach
Source: PLoS Negl Trop Dis. 2016 May 9;10(5):e0004680. doi: 10.1371/journal.pntd.0004680 (PMC4861330; doi:10.1371/journal.pntd.0004680)
Supplement: S1 Text — (DOCX) [file pntd.0004680.s010.docx]

**4-infection model equations**

System of differential equations for the 4-infection model. The parameters are equivalent to the 2-infection case. The subscripts (0000) denote the history of infection, where a zero denotes naivety to the ith serotype and a one denotes a current or prior infection. I_≥2i_ denotes all individuals with a secondary, tertiary or quaternary infection currently infectious with serotype i. This system of equations is adapted from [1].

$$\begin{matrix} \frac{dS}{dt}=\mu-\beta S(I_{1_{all}}+aI_{\geq2_{all}}+\delta)-\mu S \\ \frac{dI_{1000}}{dt}=\beta S(I_{1000}+aI_{\geq2_{1}}+\delta)-\gamma I_{1000}\mu I_{1000} \\ \frac{dI_{0100}}{dt}=\beta S(I_{0100}+aI_{\geq2_{2}}+\delta)-\gamma I_{0100}\mu I_{0100} \\ \frac{dI_{0010}}{dt}=\beta S(I_{0010}+aI_{\geq2_{1}}+\delta)-\gamma I_{0010}\mu I_{0010} \\ \frac{dI_{0001}}{dt}=\beta S(I_{0001}+aI_{\geq2_{1}}+\delta)-\gamma I_{0001}\mu I_{0001} \\ \frac{dC_{1000}}{dt}=\gamma I_{1000}-\rho I_{1000}-\mu I_{1000} \\ \frac{dC_{0100}}{dt}=\gamma I_{0100}-\rho I_{0100}-\mu I_{0100} \\ \frac{dC_{0010}}{dt}=\gamma I_{0010}-\rho I_{0010}-\mu I_{0010} \\ \frac{dC_{0001}}{dt}=\gamma I_{0001}-\rho I_{0001}-\mu I_{0001} \\ \frac{dP_{1000}}{dt}=\rho I_{1000}-\alpha\beta P_{1000}(I_{1_{i\neq1}}+aI_{\geq2_{i\neq1}}+\delta)-\mu P_{1000} \\ \frac{dP_{0100}}{dt}=\rho I_{0100}-\alpha\beta P_{0100}(I_{1_{i\neq2}}+aI_{\geq2_{i\neq2}}+\delta)-\mu P_{0100} \\ \frac{dP_{0010}}{dt}=\rho I_{0010}-\alpha\beta P_{0010}(I_{1_{i\neq3}}+aI_{\geq2_{i\neq3}}+\delta)-\mu P_{0010} \\ \frac{dP_{0001}}{dt}=\rho I_{0001}-\alpha\beta P_{0001}(I_{1_{i\neq4}}+aI_{\geq2_{i\neq4}}+\delta)-\mu P_{0001} \\ \end{matrix}$$

$$\begin{matrix} \frac{dP_{1110}}{dt}=\rho C_{1110}-\alpha\beta P_{1110}(I_{0001}+aI_{\geq2_{i=4}}+\delta)-\mu P_{1110} \\ \frac{dP_{1101}}{dt}=\rho C_{1101}-\alpha\beta P_{1101}(I_{0010}+aI_{\geq2_{i=3}}+\delta)-\mu P_{1101} \\ \frac{dP_{1011}}{dt}=\rho C_{1011}-\alpha\beta P_{1011}(I_{0100}+aI_{\geq2_{i=2}}+\delta)-\mu P_{1011} \\ \frac{dP_{0111}}{dt}=\rho C_{0111}-\alpha\beta P_{0111}(I_{1000}+aI_{\geq2_{i=1}}+\delta)-\mu P_{0111} \\ \frac{dI_{1111}}{dt}=\alpha\beta P_{1110}(I_{0001}+aI_{\geq2_{4}}+\delta)-\gamma I_{1111}-\mu I_{1111} \\ \frac{dI_{1111}}{dt}=\alpha\beta P_{1101}(I_{0010}+aI_{\geq2_{3}}+\delta)-\gamma I_{1111}-\mu I_{1111} \\ \frac{dI_{1111}}{dt}=\alpha\beta P_{1011}(I_{0100}+aI_{\geq2_{2}}+\delta)-\gamma I_{1111}-\mu I_{1111} \\ \frac{dI_{1111}}{dt}=\alpha\beta P_{0111}(I_{1000}+aI_{\geq2_{1}}+\delta)-\gamma I_{1111}-\mu I_{1111} \\ \frac{dR}{dt}=\gamma I_{1111}-\mu R \end{matrix}$$

**References**

1. Alfaro-Murillo JA, Towers S, Feng Z. A deterministic model for influenza infection with multiple strains and antigenic drift. . 2013;7: 199-211.
