# Supplementary material for: The Role of Serotype Interactions and Seasonality in Dengue Model Selection and Control: Insights from a Pattern Matching Approach
Source: PLoS Negl Trop Dis. 2016 May 9;10(5):e0004680. doi: 10.1371/journal.pntd.0004680 (PMC4861330; doi:10.1371/journal.pntd.0004680)
Supplement: S2 Text — (DOCX) [file pntd.0004680.s011.docx]

## Appendix S1: Proof R_0_

We use the next generation matrix to compute the basic reproduction number (R_0_) associated with the disease-free equilibrium ^[1-3]^. To obtain the disease-free equilibrium, we assume all parameters are constant over time and thus ignore the effect of seasonal forcing. The disease-free equilibrium for the system $(S,I_{i=1-n},C_{i=1-n},P_{i=1-n},I_{\begin{matrix} i=1-n \\ j=1-n\neq i \end{matrix}},R)$ , where n is the number of serotypes equals$E_{0}=(1,0,0,0,0,0)$. For simplicity, we show the derivation for n=2 serotypes, which gives the same result as larger serotype systems ^[4]^. From the infection terms $(I_{i=1-n},I_{\begin{matrix} i=1-n \\ j=1-n\neq i \end{matrix}})$ , with n=2:

$$\begin{matrix} \frac{dI_{1}}{dt}=\beta_{t}S(I_{1}+aI_{21}+\delta)-\gamma I_{1}-\mu I_{1} \\ \frac{dI_{2}}{dt}=\beta_{t}S(I_{2}+aI_{12}+\delta)-\gamma I_{2}-\mu I_{2} \\ \frac{dI_{12}}{dt}=\alpha\beta_{t}P_{1}(I_{2}+aI_{12}+\delta)-\gamma I_{12}-\mu I_{12} \\ \frac{dI_{21}}{dt}=\alpha\beta_{t}P_{2}(I_{1}+aI_{21}+\delta)-\gamma I_{21}-\mu I_{21} \end{matrix}$$

we can derive the non-negative matrix, F, which represents the rate of appearance of new infections at each infectious stage and, at the disease-fee equilibrium, is denoted as:

$$F=\left(\begin{matrix} \beta& 0 & 0 & \beta a \\ 0 & \beta& \beta a & 0 \\ 0 & 0 & 0 & 0 \\ 0 & 0 & 0 & 0 \end{matrix}\right)$$

And the rate of change by all other means, at the disease-free equilibrium is defined as:

$$V=\left(\begin{matrix} \gamma+\mu& 0 & 0 & 0 \\ 0 & \gamma+\mu& 0 & 0 \\ 0 & 0 & \gamma+\mu& 0 \\ 0 & 0 & 0 & \gamma+\mu\end{matrix}\right)$$

With its inverse being:

$$V^{-1}=\left(\begin{matrix} \frac{1}{\gamma+\mu} & 0 & 0 & 0 \\ 0 & \frac{1}{\gamma+\mu} & 0 & 0 \\ 0 & 0 & \frac{1}{\gamma+\mu} & 0 \\ 0 & 0 & 0 & \frac{1}{\gamma+\mu} \end{matrix}\right)$$

The basic reproduction number is defined as the largest eigenvalue of the matrix FV^-1^, thus

$$0=\det(FV^{-1}-\lambda I)=\det\left(\begin{matrix} \frac{\beta}{\gamma-\mu}-\lambda& 0 & 0 & \frac{\beta a}{\gamma-\mu}-\lambda\\ 0 & \frac{\beta}{\gamma-\mu}-\lambda& \frac{\beta a}{\gamma-\mu}-\lambda& 0 \\ 0 & 0 & -\lambda& 0 \\ 0 & 0 & 0 & -\lambda\end{matrix}\right)$$

where I is the identity matrix. Solving the determinant of this matrix leads to the basic reproduction number being:

$$R_{0}=\frac{\beta}{\gamma+\mu}$$

Where the disease-free equilibrium is stable for values of R_0_<1 and unstable for values of R_0_>1. Mark that the stability of the disease-free equilibrium is not dependent on the ADE or cross-immunity. Because symmetry between the strains is assumed (α,a,β_0_, β_1_, γ and τ are equal for all strains), R_0_ is equal for all strains.

## References

1. Diekmann O, Heesterbeek J, Metz J. (1990) On the definition and the computation of the basic reproduction ratio R 0 in models for infectious diseases in heterogeneous populations. J Math Biol 28: 365-382.

2. Van den Driessche P, Watmough J. (2002) Reproduction numbers and sub-threshold endemic equilibria for compartmental models of disease transmission. Math Biosci 180: 29-48.

3. Diekmann O, Heesterbeek JAP. (2000) Mathematical epidemiology of infectious diseases: Model building, analysis and interpretation. : Wiley.

4. Billings L, Schwartz IB, Shaw LB, McCrary M, Burke DS, et al. (2007) Instabilities in multiserotype disease models with antibody-dependent enhancement. J Theor Biol 246: 18-27.
